# Supplementary material for: REDCRAFT: A computational platform using residual dipolar coupling NMR data for determining structures of perdeuterated proteins in solution
Source: PLoS Comput Biol. 2021 Feb 1;17(2):e1008060. doi: 10.1371/journal.pcbi.1008060 (PMC7877757; doi:10.1371/journal.pcbi.1008060)
Supplement: S1 Table — (DOCX) [file pcbi.1008060.s001.docx]

| Structure | **6E4J** | **6NS8** |
| --- | --- | --- |
| ^15^N-^1^H RDCs | no | 217 |
| Backbone Phi/Psi Restraints^a^ | 132 | 132 |
| ^15^N NOESY peaks | 1120 | 1074 |
| ^13^C NOESY peaks | 3460 | 3295 |
| Total NOESY peaks | 4580 | 4369 |
| Assigned by CYANA | 4052 | 4046 |
| CYANA Target Function | 0.81 ± 0.03 | 1.15 ± 0.07 |

^a^ Determined by TALOS_N
