# Supplementary material for: REDCRAFT: A computational platform using residual dipolar coupling NMR data for determining structures of perdeuterated proteins in solution
Source: PLoS Comput Biol. 2021 Feb 1;17(2):e1008060. doi: 10.1371/journal.pcbi.1008060 (PMC7877757; doi:10.1371/journal.pcbi.1008060)
Supplement: S2 Table — (DOCX) [file pcbi.1008060.s002.docx]

|  | **6E4J** | **6NS8** |
| --- | --- | --- |
| **Summary of conformationally-restricting**  **experimental restraints ^a^** |  |  |
| *NOE-based distance restraints* |  |  |
| Total | 2372 | 2328 |
| intra-residue [i = j] | 490 | 479 |
| sequential [\| i - j \| = 1] | 574 | 551 |
| medium range [1 < \| i - j \| < 5] | 770 | 752 |
| long range [\| i - j \| ≥ 5] | 538 | 546 |
| NOE constraints per restrained residue ^b^ | 32.9 | 32.3 |
| *Hydrogen bond restraints:* |  |  |
| Total | 70 | 74 |
| long range [\| i - j \| ≥ 5] | 0 | 0 |
| ***RDC restraints*** |  | **217** |
| ***Number of alignment media*** |  | **2** |
| *Dihedral-angle restraints:* | 132 | 132 |
| *Total number of restricting restraints ^b^* | 2574 | 2534 |
| *Total number of restricting restraints per restrained residue ^b^* | 35.8 | 35.2 |
| *Restricting long-range restraints per restrained residue ^b^* | 7.5 | 7.6 |
| **Total structures computed** | 100 | 100 |
| **Number of structures reported** | 20 | 20 |
|  |  |  |
| **Residual constraint violations ^a,c^** |  |  |
| *Distance violations / structure* |  |  |
| 0.1 - 0.2 Å | 4.5 | 4.8 |
| 0.2 - 0.5 Å | 1.4 | 0.55 |
| > 0.5 Å | 0 | 0 |
| RMS of distance violation / constraint | 0.01 Å | 0.01 Å |
| Maximum distance violation ^d^ | 0.37 Å | 0.44 Å |
| *Dihedral angle violations / structure* |  |  |
| 1 - 10 ° | 3.6 | 6.95 |
| > 10 ° | 0 | 0 |
| RMS of dihedral angle violation / constraint | 0.41 ° | 0.78 ° |
| Maximum dihedral angle violation^d^ | 8.20 ° | 9.10 ° |
| Q-factor^e^  Medium M1  Medium M3 | 0.339 ± 0.020  0.319 ± 0.031 | 0.275 ± 0.015  0.279 ± 0.028 |
| **RPF scores** |  |  |
| Recall | 0.966 | 0.965 |
| Precision | 0.975 | 0.977 |
| F measure | 0.971 | 0.971 |
| DP score | 0.905 | 0.905 |
| **RMSD Values** |  |  |
| all |  |  |
| All backbone atoms | 0.5 Å | 0.5 Å |
| All heavy atoms | 0.8 Å | 0.9 Å |
| well-defined ^f^ |  |  |
| All backbone atoms | 0.5 Å | 0.5 Å |
| All heavy atoms | 0.8 Å | 0.8 Å |
| **Structure Quality Factors - overall statistics** (Raw/Z- score) |  |  |
| Procheck G-factor ^f^ (phi / psi only) | 0.27 / 1.38 | 0.09 / 0.67 |
| Procheck G-factor ^f^ (all dihedral angles) | 0.12 / 0.71 | 0.07 / 0.41 |
| Verify3D | 0.46 / 0.00 | 0.40 / -0.96 |
| ProsaII | 1.19 / 2.23 | 1.18 / 2.19 |
| MolProbity clashscore | 18.68 / -1.68 | 14.41 / -0.95 |
| **Ramachandran Plot Summary from Procheck ^f^** |  |  |
| Most favored regions | 97.0% | 97.1% |
| Additionally allowed regions | 3.0% | 2.9% |
| Generously allowed regions | 0.0% | 0.0% |
| Disallowed regions | 0.0% | 0.0% |
| **Ramachandran Plot Statistics from Richardson's lab** |  |  |
| Most favored regions | 99.8% | 97.5% |
| Allowed regions | 0.2% | 2.5% |
| Disallowed regions | 0% | 0% |

^a^Analyzed for residues 1 to 72. Generated using PSVS 1.5
^b^ There are 72 residues with conformationally restricting constraints
^c^ Calculated for all constraints for the given residues, using sum over r^-6
^d^ Largest restraint violation among all the reported structures

^e^  Analyzed with RedCatr
^f^ Well-defined residues selected based on: Dihedral angle order parameter, with S(phi)+S(psi)>=1.8. Well-defined residue range: 2A-72A
^g^ With respect to mean and standard deviation for a set of 252 X-ray structures < 500 residues, of resolution <= 1.80 Å, R-factor <= 0.25 and R-free <= 0.28; a positive value indicates a 'better' score.
