# Supplementary figures and images for: REDCRAFT: A computational platform using residual dipolar coupling NMR data for determining structures of perdeuterated proteins in solution
Source: PLoS Comput Biol. 2021 Feb 1;17(2):e1008060. doi: 10.1371/journal.pcbi.1008060 (PMC7877757; doi:10.1371/journal.pcbi.1008060)

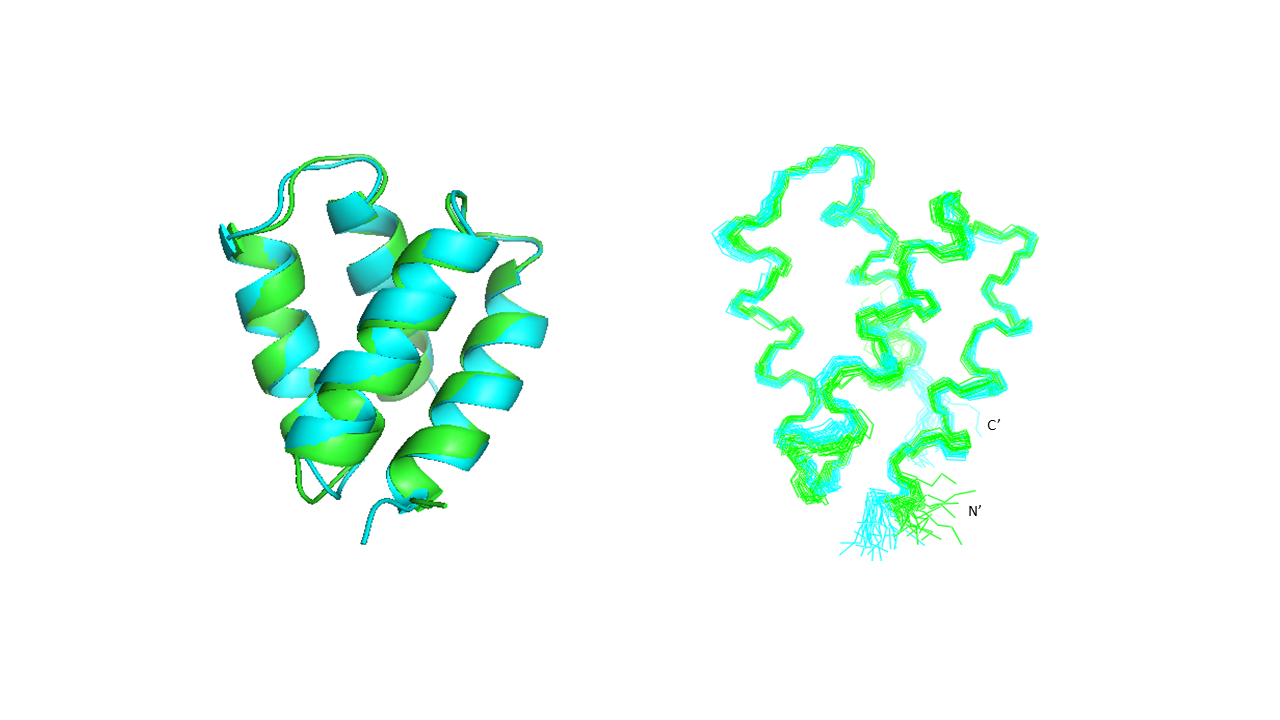

Supplement: S1 Fig — The overall RMSD is within 0.6 Å. (TIF) [file pcbi.1008060.s003.tif]

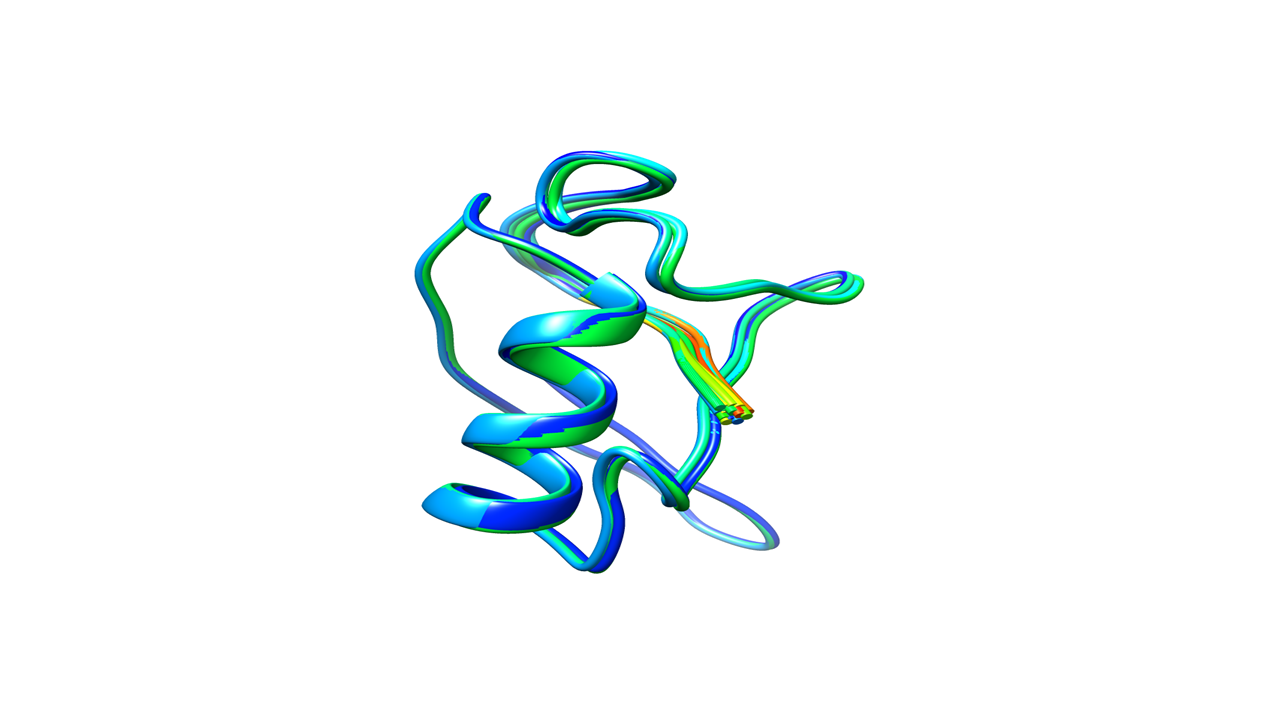

Supplement: S2 Fig — The structural ensemble exhibit pairwise BB-RMSD of less than 0.5 Å. (TIF) [file pcbi.1008060.s004.tif]

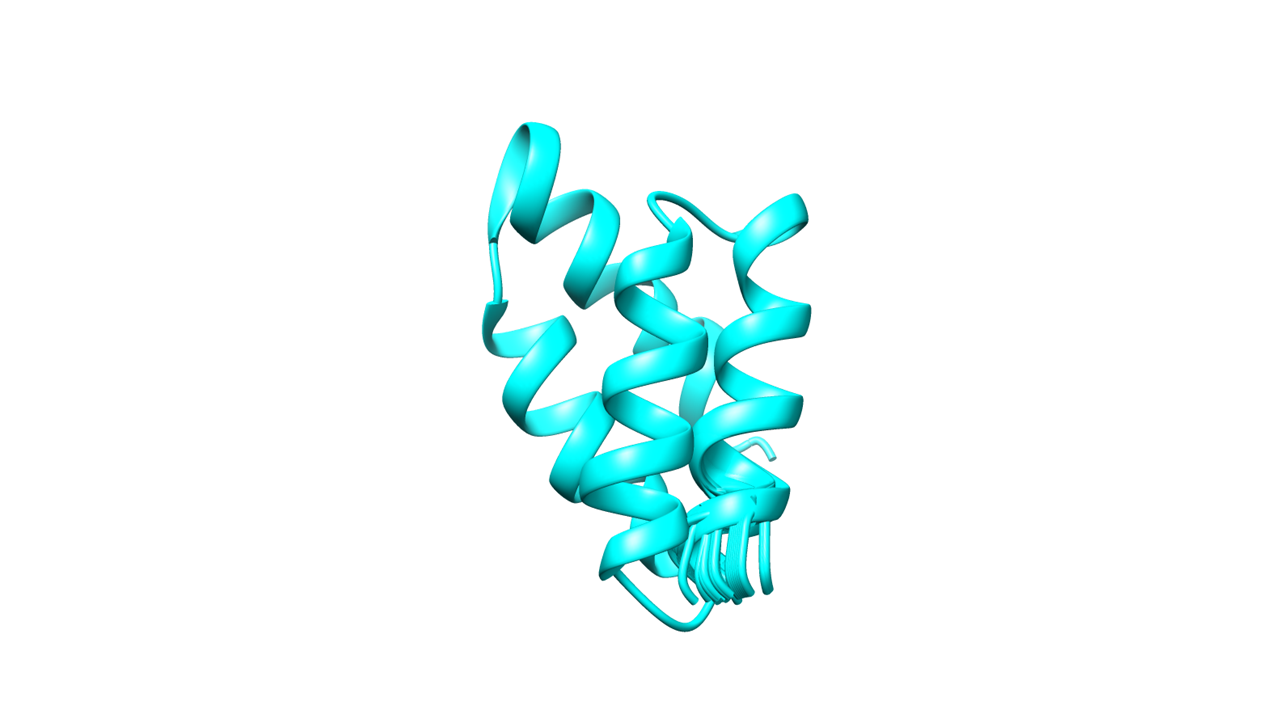

Supplement: S3 Fig — The structural ensemble exhibit pairwise BB-RMSD of less than 1.005 Å. (TIF) [file pcbi.1008060.s005.tif]
