# Supplementary material for: REDCRAFT: A computational platform using residual dipolar coupling NMR data for determining structures of perdeuterated proteins in solution
Source: PLoS Comput Biol. 2021 Feb 1;17(2):e1008060. doi: 10.1371/journal.pcbi.1008060 (PMC7877757; doi:10.1371/journal.pcbi.1008060)
Supplement: Attachment — Submitted filename: cole_valafar_review.pdf [file pcbi.1008060.s006.pdf]

The manuscript by Cole, Daighma, Liu, Montelionoe and Valafar talks about solving protein structures based on residual dipolar couplings (RDCs) alone, with no recourse to traditional NOE data.

This appears to be an attractive route, given that collection and analyses of NOEs is the bottleneck in the conventional structure-solving routine. The authors present a small set of structures that have been solved by means of their program REDCRAFT using RDCs alone. The set includes a *de novo* structure of small archaeal protein PF2048.1, which has no sequence homologues in the PDB. The ability of REDCRAFT to solve structures is truly impressive (especially given an early skepticism in the field). It would definitely be useful to make bio-NMR community aware of these remarkable capabilities.

At the same time, as argued below, there is probably a good reason why people do not solve structures based on RDCs alone, but rather prefer to rely on NOEs (and/or other data such as chemical shifts, PREs, etc.). It should be pointed out that the manuscript is unusual in that it reads a bit like an advertisement of REDCRAFT (which is not a new software and has been quite extensively covered by the authors before).

On balance, I suggest that the paper should be published – after revising some of the more general aspects of the discussion + addressing a number technical glitches.

1. I would argue that the advantage of RDC route over the more traditional routes is largely fictional. Indeed, one needs to make 2-3 aligned samples. This is a challenge – for a given protein, some alignment media would work, while others would not (the sample would crash out, etc.). This means 4-6 sample preps, only half of which typically proves to be successful. This also means that the lab should be equipped with the materials and tools to make such multiple aligned samples. After that one needs to record N-HN RDCs (which is not difficult), but additionally some longer-range RDCs, which normally requires several 3D experiments.

Clearly, we are talking here about the investment of minimum 1 month, and more likely several months in the case of an average graduate student. On the other hand, NOESY experiment does not require any samples beyond the one that already has been used for resonance assignment, the measurement itself takes several days to a week, while data analysis is largely automated nowadays and does not take much time either. So why should one choose the RDC-based route?

2. To further elaborate on this point, both RDC and NOE data contain errors due to misassignments, overlaps, etc. The NOE-based structure-solving procedure is very stable with respect to a small number of erroneous data. What about the RDCs? This aspect is not addressed in the paper at all (how to handle the outliers) – and I suspect that the procedure can be derailed by a small number of outliers.

What happens when there is a gap of several residues – no RDC data from a stretch of several contiguous residues (or very few RDC data). This would seem to derail the structure-building

procedure because we would lack the information about the “translation” of the peptide chain (RDCs only carry the orientational information). The authors should explain this better instead of simply saying that “any existing gaps of less than 6 amino acids can easily be filled during the process of structural refinement”(?)

Is it possible to build “mirror structures” given the degeneracies that are present in the RDCs (at least for local fragments, where few RDCs are available because of spectral overlaps, etc.)?

3. The effect of dynamics. Per-residue RDCs can be almost arbitrarily scaled by dynamics. As pointed out by the authors, having an extensive set of experimental RDCs (like 10 pieces of data per residue), one can separate “dynamics” from “structure”. How is it done in the current treatment, though? It seems that the local dynamics is ignored in this manuscript – isn’t there a price to be paid in terms of accuracy?

4. One should also note that REDCRAFT approach only generates backbone folds (i.e. no side chains). Even though the authors mention that there are computational tools to rebuild side chains, this is a major handicap – the resulting structure is essentially a partial model.

5. The accuracy is also not great. Aside from small model proteins, the accuracy seems to be ca. 2 Å. This is, of course, not suitable for any precision work (such as rational drug design) – especially in the absence of experimentally determined side-chain coordinates as mentioned above. On the other hand, to be fair, the accuracy of conventional NMR structures is also not great (when assessed against X-ray structures) – the authors may want to discuss this aspect as well.

The authors say “Although NMR studies may in general be more time consuming and costly than X-ray crystallography”. They should also mention less accurate – this is a major consideration from consumers’ standpoint.

6. Generally, my feeling is that the authors oversell the advantages of RDCs and of their strategy.

For instance, they say: “Indeed, in the 10 ns – 1 s timescale window, RDCs are the most sensitive of NMR parameters(32)”. This cannot be true. While RDCs are the only parameters with some sensitivity to dynamics in the 10 ns – 10 us range, this information is very difficult to get at. There has been a major debate in the field, involving Bax, Clore, Griesinger, Bruschweiler, Torchia, Tolamn and others about getting a handle on dynamics using RDCs – precisely for the reason that this is such a muddy business. In the 10 us – 1 s range there are many NMR experiments that provide a much more clear-cut answers on dynamics.

It would be really helpful if the authors present a more balanced view of their RDC-based method instead of a sales-pitch narration.

7. The authors also seem to be wedded to the idea of using RDCs alone. They occasionally use XPLOR-NIH for structure refinement, which actually makes a big difference (see Tab. 2). But they

seek to downplay it, e.g. “Aside from completion of the missing residues, these minimizations normally resulted in structural variation of less than 0.5A”.

Why insist on using RDCs alone? It would be a good idea to exploit force fields (physics-based or knowledge-based such as ROSETTA). It would be a good idea to throw in other NMR data such as chemical shifts – those are free, they do not require any special measurements (since the assignment is needed anyways). The authors say: “[Certain studies] resorted to the use of other experimental data such as NOEs, dihedral restraints and hydrogen bond restraints (21, 65, 66) for successful structure determination. Both of these approaches nullify the advantages of using RDCs (minimal data, reduced cost)”. This is, generally speaking, not true. Dihedral angles derived from chemical shifts (TALOS) do not cost anything extra. PREs cost relatively little and are quite informative. State of the art force fields are cost-free. At the same time, as we have discussed above, RDCs are actually rather costly.

As a methodological point it is interesting to demonstrate that structures (or rather backbone folds) can be successfully obtained by using RDCs alone. But the way into the future is to (optimally) combine RDCs with other pieces of experimental data and with computer modeling.

8. The manuscript is structured in a bit of an awkward way. Over the first 23 pages it talks about the unique advantages of REDCRAFT without actually saying what’s the origin of these advantages. The material at p. 23, which talks about REDCRAFT algorithm, rather belongs to an introduction. Also it needs to be extended and qualified to describe all the limitations mentioned above.

In the Materials & Methods, the authors say “In this report we will demonstrate the success of REDCRAFT”, but that’s actually near the end of the paper.

9. In some ways, Fig. 5 demonstrates the advantages of NOEs over RDCs – not the other way around. Definitely much better radius of convergence. Also the authors need to add a panel to this graph, zooming in on the range 0-0.05 along the y-axis. Only with this zoom-in we can form an impression about the ability of NOEs to guide structure-optimization procedure toward the global minimum. The existing plot is misleading in this sense simply because of the plotting scale.

11. The description of the REDCRAFT does not explain very clearly if there are any tunable parameters that need to be adjusted prior to REDCRAFT calculations (although it is mentioned that some modules have been disabled). If success of structure optimization depends on certain program settings, this is very important to mention. If not, this needs to be mentioned too.

Some more technical issues:

12. In Tab. 1 and the surrounding discussion, the authors use values (like 1.189), ranges of values (1.9 - 2.5), lists of values (0.098, 0.12) and lists of ranges (0.01 - 0.046, 0.01 - 0.038, 0.01 - 0.026). They never explain the meaning of this notational diversity. I suppose it has to do with multiple conformers in the NMR structures and multiple alignment media.

13. Tab. 1 and Fig. 1 do not quite agree. According to Tab. 1, it is x-ray structure which is not always available (panel D in the plot), not an NMR structure as implied in the figure caption. It is unclear why panel E in the plot misses the NMR (green) structure.

14. “By starting the calculation for the entire protein, the algorithm must contend with the maximum level of complexity from the start, which transforms the problem into a global optimization”. This may be a problem for RDCs, but it is not a problem for NOEs, which handle such global optimization task just fine.

15. “Sequence specific backbone and side-chain NMR resonance assignments were determined using standard triple-resonance NMR experiments (Table S2)”. Table S2 does not say anything about the assignment experiments. Nowhere in the text have the authors mentioned the experiments used to measure RDCs in PF2048.1 (although protein expression and purification are described in some detail, as well as the preparation of aligned samples).

15. Wording:

“due to their planarity with one another” – due to their co-planarity
“both structures … exhibited high quality structures”
“large proteins (as large as 573)” – 573 residues
“RECRAFT’s approach”
“aqueous proteins”
“an excessive number of RDCs than necessary” – excessive number or more than necessary
“computational models … concluded the helical nature … and resulted in an ensemble of structures”
One and the same program is referred as RPF and RFP
