# Supplementary material for: REDCRAFT: A computational platform using residual dipolar coupling NMR data for determining structures of perdeuterated proteins in solution
Source: PLoS Comput Biol. 2021 Feb 1;17(2):e1008060. doi: 10.1371/journal.pcbi.1008060 (PMC7877757; doi:10.1371/journal.pcbi.1008060)
Supplement: Attachment — Submitted filename: cole_valafar_revised_review.pdf [file pcbi.1008060.s008.pdf]

The manuscript by Cole, Daigham, Liu, Montelione and Valafar talks about solving protein structures based on residual dipolar couplings (RDCs).

The text of the manuscript has been thoroughly revised. Following the revisions, I have no problem with the structure of the paper and its main message. I would like to thank the authors for their thoughtful and constructive approach to my previous critique.

Still, there are a couple of fairly significant issues that I would like to raise. In addition, there are quite a few minor glitches in the text. It appears that no one actually proof-read the text before resubmitting it to the journal, which is somewhat disappointing. In any event, the paper can now be published after some minor revisions. I trust that the authors can take care of the remaining issues (there is no need for me to re-review this manuscript).

1. Response 2.5. "In cases where significant differences are observed between NOE-based models and the corresponding X-ray structures, the X-ray structure coordinates are often found to be inconsistent with the NOE data. Hence, the X-ray crystal structure may be very accurate relative to what is present in the crystal, but not accurately reflect the dominant structure in solution."

I believe this is a misconception. X-ray coordinates provide a far more accurate representation of protein structure *in solution* than NMR coordinates. To make sure that this is so, let us take a random globular protein, where both X-ray structure (with resolution 1.8 A or better) and NOE-based structure are available. Let us consider proteins other than ubiquitin or GB1 (for which there are some extraordinarily accurate NOE structures), but a run-of-the-mill protein. Then let us fit a set of experimental *solution-state* RDCs to the X-ray structure and, separately, to the NOE-based structure. There is little doubt that X-ray structure should produce a much better *Q*-factor than NOE-based structure – thus signifying that X-ray structure is a much better model for protein in solution. One can also repeat the same exercise with *J*-couplings, chemical shifts and other solution-state observables.

2. Let me use X-ray crystallography as an example to make another point. Conventional protocol to solve a crystal structure ends at the refinement step, where one obtains, say, crystallographic R of 0.21 and R_{free} of 0.24. In principle, one could drive the refinement further and achieve, say, R=0.08. But there is a price to pay, since R_{free} shoots to something like 0.36 (one can find some examples of this behavior in the early literature). This type of a situation is not good – in the case of (R=0.08, R_{free}=0.36) the coordinates are actually overtightened and have lower quality than in the case of (R=0.21, R_{free}=0.24). I imagine that something like this also happens with the RDC-based refinement. When the authors obtain Q factors of 0.06 to 0.09 – these are likely overtightened structures of somewhat inferior quality. It would be good to investigate this aspect using a system with some redundancy in the measured RDC dataset, so that Q_{free} can be formed and inspected.

3. Abstract. "Here, we describe the new features of the protein structure modeling program REDCRAFT and focus on the new Adaptive Decimation (AD) feature. The AD plays a critical role

in improving the robustness of REDCRAFT to missing or noisy data, while allowing structure determination of larger proteins from less data".

This leads one to think that AD is a centerpiece of the paper. After that it is barely mentioned at all throughout the rest of the paper (Introduction, Results, Discussion) – all the way to the Algorithms and Methods section, where it is actually described quite nicely. This produces a bot of a strange impression. The authors may want to somehow correct this – either briefly describe the AD method in the Introduction or, alternatively, tone down the abstract.

4. Line 67. "Despite the changes that NMR spectroscopy has overcome over the years". Changes that NMR spectroscopy underwent (or challenges that NMR spectroscopy has overcome).

5. Lines 92 and 98. "continued use of the conventional optimization techniques", "continue to rely on the traditional optimization techniques" – repetitive.

6. The authors constantly use the term "legacy software" for NMR-structure-solving programs. The term "legacy" means "no longer under active development". Do the authors imply that none of NMR-structure-solving programs are under active development anymore…?

7. Line 112 and throughout the text. "…can be obtained from perdeuterated proteins, namely backbone C'-N, N-H^N, and C'-H RDCs". Does the latter mean C'-H^N? This inconsistency persists throughout the text (and in Tab. 4 one can also find N-H notation alongside with N-H^N, same in line 649; also one comes across H-C_α, which appears distinct from H_α-C_α).

8. Line 163. "a Q-factors"

9. Line 177. "An example of the convergence of the top 50 ensemble structures resulting from REDCRAFT calculation for GB1 is shown in Supplemental Fig S2. These represent the top 50 structures calculated for GB1 using this set of RDC data". The second sentence is redundant.

10. Line 186. "these RDC data was reduced"

11. Table 1 and elsewhere. There are rmsd values reported with three digits, e.g. 1.121 Å. Should be rounded to 1.12.

12. Lines 247, 250. "Van der Waals" should be van der Waals.

13. Line 249. "The Q-factor RDCs" should be "The Q-factor for RDCs"

14. Line 256. "An example of the convergence of the top 50 ensemble structures resulting from REDCRAFT calculation for PF2048.1 is shown in Supplemental Fig S3. These represent the top 50 structures calculated for PF2048.1 using this set of RDC data". The second sentence seems redundant.

15. Line 305. "Structure calculation of large proteins by NMR spectroscopy is facilitated through perdeuteration of the sample protein suppress nuclear relaxation pathways…". Should be "perdeuteration of the sample protein, which suppresses"

16. Line 322. "This is a significant achievement since in most cases, since such proteins must be perdeuterated …"

17. Line 350. "Structural elucidation of proteins from RDCs using REDCRAFT has other pragmatic advantages. For instance, characterization of protein structure does not have to be restricted to the entire protein". You can solve protein fragments instead of an entire structure using any data (including NOEs) – this is not a "pragmatic advantage" of RDCs.

18. Line 429. "Therefore, NOEs become relatively insensitive to structural variation when less than 5 Å from the native structure." The plot suggests more like 2 Å. Indeed, if it would have been 5 Å, then NOE-based structures would have an accuracy of about 5 Å. This is not the case – their true accuracy is more like 2 Å. For your information, that's much worse than the accuracy of a reasonable X-ray structure with a resolution of, says, 1.8 Å – the accuracy in this case amounts to about 0.3 Å (please see my comment #1).

19. Line 492. "Amide sides" should be "sites"

20. Section Evaluation, line 520. "Our evaluation of REDCRAFT was conducted in three phases…". The order of the "phases" discussed in this section is not the same as in the actual text.

21. Both Tables 4 and 5 can be easily relegated to the SI.

22. Line 610. "The NxSC1 was pooled…". Why it is called NxSC1 here instead of PF2048.1?

23. Line 629. "RDC data suing the software package REDCAT". Those litigious RDC data…
