# Supplementary material for: REDCRAFT: A computational platform using residual dipolar coupling NMR data for determining structures of perdeuterated proteins in solution
Source: PLoS Comput Biol. 2021 Feb 1;17(2):e1008060. doi: 10.1371/journal.pcbi.1008060 (PMC7877757; doi:10.1371/journal.pcbi.1008060)
Supplement: Attachment — Submitted filename: ResponseToReviewers.Final.docx [file pcbi.1008060.s007.docx]

# **Reviewer 1**

The authors present an update to their REDCRAFT software, which is designed to calculate the structure of proteins from residual dipolar coupling data. Previous iterations of REDCRAFT have been described in several publications: J Magn Reson 2008, J Magn Reson 2010, J Biomol NMR 2014, and JCTC 2016. From what I could gather, this manuscript presents the largest benchmark of REDCRAFT to date, which is commendable. I think, however, that the manuscript could benefit from an extensive reorganization to highlight the novel features of REDCRAFT. This current version reads often as if the algorithm has never been described. In addition, I could not find a mention to exactly what new features the authors included in the latest version of REDCRAFT. The authors also fail to provide a URL for either the source code or the binaries of the software. This manuscript seems to be a benchmark and demo of REDCRAFT's abilities on a variety of proteins. While I find this important, new developments in NMR software should be encouraged, I cannot recommend the article for publication without major revisions. In particular, the authors should trim the contents of the manuscript to focus on a simpler message: what is new in REDCRAFT. Otherwise, the authors should just say, here's our code tested in a large benchmark and compared to traditional methods. Finally, I believe that there is room for some extra experiments (all computational) that could bring some novelty and added value to the work.

Response: We thank this reviewer and their detailed feedback. We have incorporated nearly all the suggested changes and as a result, the substantially revised document has improved both in the content and the related discussions.

More specifically, we have addressed the general comments of this reviewer by first clarifying the new features of REDCRAFT (Dec 2019 latest release), and highlighting the new Adaptive Decimation feature that has played a critical role in improving the resilience of REDCRAFT to missing or noisy data, while allowing for structure determination of larger proteins with less data. A summary of new and novel features is presented on page 3, lines 105-111 and pages 11-12, lines 370-425 of the revised manuscript. A URL for accessing source code and binaries is included on lines 108 and 381.

Below are my comments on each section of the manuscript that I hope can help the authors in their revisions:

Comment 1.1: From the abstract and author summary, I cannot find what novel features have been added to this version of the software. The authors should make it very clear what is new in this version and why it matters to the users.

Response 1.1: We have modified the abstract and the following text to more clearly state the new features of REDCRAFT, Adaptive Decimation in particular. More specifically the following segments have been added to the Abstract and the Algorithms and Methods section, respectively:

Here, we describe the new features of the protein structure modeling program REDCRAFT and focus on the new Adaptive Decimation (AD) feature. The AD plays a critical role in improving the robustness of REDCRAFT to missing or noisy data, while allowing structure determination of larger proteins from less data.

REDCRAFT was previously introduced^12^ to provide a more robust means of structure determination and detangling of structural information from internal dynamics^43,76^. The most recent version of REDCRAFT (released in Dec. 2019 and available from: https://bitbucket.org/hvalafar/redcraft/src/master/) aims to improve the usability of the software through the inclusion of a Graphical User Interface, compliance with NEF data exchange format^77^, and inclusion of improved user documentation. Other software engineering design improvements allow for universal portability and usability in MAC, Linux, and Windows, and improved maintainability. In addition, the newest version of REDCRAFT integrates existing dihedral information either from experimental sources such as TALOS^78^, or knowledge bases such as PDB^37^ or PDBMine^79^. In this report, we present the Adaptive Decimation feature that has enabled the use of less RDC data to determine 3D structures of larger proteins. We will begin our discussion of REDCRAFT with some of the key features that enable unique analysis of RDCs and extend the discussion to introduce Adaptive Decimation.

Comment 1.2: As much as I like NMR, of the structures deposited in the RCSB PDB in the last 5 years, 50127 were determined by X-ray crystallography, 4468 by EM, and 2074 by NMR (both ssNMR and solution). I would change the very first sentence in the abstract to state that there are 'three primary experimental means of characterizing macromolecular structures'.

Response 1.2: Thank you for this helpful comment. This change has been incorporated in the Abstract.

Comment 1.3: I would also note that structure determination with/from RDC data is not recent. There are many papers from the early 2000s (so, nearly 20 years ago) describing applications and methods.

Response1.3: We have modified the text to either exclude temporal reference, or include terms such as “recent decades”, “relatively recent” etc. to address this issue. Our use of the term “Recent” was relative to the history of NMR.

Comment 1.4: On line 40, the authors write that structure determination with RDCs is limited to small proteins 'using RDC data from many alignment media (>3) that cannot be collected from large proteins'. Then, on line 45, they state that REDCRAFT can be applied to large proteins using simulated RDC data from perdeuterated proteins. So, can we use RDCs for large proteins or not?

Response 1.4: This portion of the abstract has been replaced with new material to reflect the new features of REDCRAFT and therefore present a clearer description of the content. The general message that we would like to convey to the readers is the ability of REDCRAFT to generate accurate model of larger perdeuterated proteins, where only limited NOE data due to few sidechain resonance assignments are available. Other comparable RDC-based methods have only been applied to smaller proteins (< 10 kDa), but the latest version of REDCRAFT can be applied to larger systems, as demonstrated in this work. We have emphasized this difference in numerous places including the REDCRAFT section. Below are some snippets of related sentences:

In this report, we present the Adaptive Decimation feature that has enabled the use of less RDC data to study larger proteins.

The Adaptive Decimation allows for an appropriately and automatically adjusted decimation threshold in order to remain effective at all stages of structure determination. A more effective decimation process will enable recovery from more erroneous or missing RDC data as demonstrated recently. In this report we demonstrate the application of Adaptive Decimation in structure calculation of proteins as large as 573 residues with a limited set of RDC data that can be obtained for perdeuterated proteins.

Comment 1.5: On line 72, if you are going to be as specific as '11.649', then do not use 'nearly'. I'd say 'nearly 12.000'.

Response 1.5: We have used the term “nearly” considering the rapid changing contents of the PDB database. We anticipate by the time this manuscript is available to the readers, the content will no longer be exact. Nevertheless, we have modified this number as suggested.

Comment 1.6: Regarding line 75, I would argue that NMR is not determining structures in their 'native' environment. The temperatures at which structures are determined and the concentrations used in the sample preparation are hardly comparable to 'native' conditions. Please remove the word 'native', as it induces the reader in error.

Response 1.6: We have modified the text to read as below to state that NMR studies allow the study of dynamical behavior of some proteins.

they (solution NMR studies) provide the unique benefit of observing macromolecules in an aqueous state closer to their native state, which provide a better understanding of molecular interactions and internal dynamics at various timescales and resolutions.

Comment 1.7: On lines 78-80, I would add molecular dynamics simulations to the list of methods used by NMR software to produce structures. CNS and XPLOR-NIH both use MD for example. I would also argue that not having new methods isn't necessarily bad. Using old forcefields and not refining structures in explicit solvent, that I'd agree with that it's bad!

Response 1.7: We agree. We have added “restrained molecular dynamics and simulated annealing” to the list of methods used by NMR software to produce structures.

Comment 1.8: On line 91, I disagree that the main limitation for studying large proteins with NMR is on the computational side, or with costs. Rather, in my experience, it's more to do with the spectral crowding and the inability to assign peaks productively.

Response 1.8: We fully agree with this reviewer’s point that the spectral crowding and general spectral quality are primary limitations on the experimental side of NMR spectroscopy. Our main assertion is that improved methods of data analysis can potentially compensate for the fewer available experimental restraints. Therefore, while experimental NMR spectroscopy is making advances, there should exists parallel advances in computational analysis methods. The manuscript has been modified on page 9 to address this point, as follows:

Structure calculation of large proteins by NMR spectroscopy is facilitated through perdeuteration of the sample protein to suppress nuclear relaxation pathways and provide sharper linewidths. Exchangeable amide sites are reprotonated by exchange, allowing ^1^H-detected heteronuclear NMR studies, including some types of RDC measurements. However, the general absence of protons other than amide protons limits the kinds of RDC data that can be measured.

Comment 1.9: I need a citation for the sentence at lines 92-93: RDCs as a promising alternative to NOEs. By the way, there's a typo in Residual on that sentence.

Response 1.9: Thank you. The correction has been made and the citation has been added.

Comment 1.10: On line 107, the authors state that 'all the existing RDC-based structure determination approaches [...]', but wouldn't that include REDCRAFT, which was first published in 2008?

Response 1.10: Thank you for this useful comment - this section of text is deleted in the revised manuscript. The previously published methods have been discussed in the RDC and REDCRAFT sections. In the REDCRAFT section, we have clearly stated the previous appearance of REDCRAFT and cited the related work. We feel that keeping the citations related to REDCRAFT separate from other methods is appropriate since REDCRAFT is the direct topic of the discussion. Below are some examples of the changes:

Other specialized software packages^8,12,18-25^ have contributed to the advancement of RDC data analysis in order to provide a more effective path to structure determination from RDC data.

REDCRAFT was previously introduced^12^ to provide a more robust means of structure determination and detangling of structural information from internal dynamics^43,76^. The most recent version of REDCRAFT (released in Dec. 2019 and available from: https://bitbucket.org/hvalafar/redcraft/src/master/) aims to improve the usability of the software through the inclusion of a Graphical User Interface, compliance with NEF data exchange format^77^, and inclusion of improved user documentation.

Comment 1.11: On line 113, as in other parts of the manuscript, the authors refer to their search/optimization strategy as 'novel', but I have read the same exact strategy in their 2008 paper. Please remove 'novel'.

Response 1.11: Our use of the adjective “novel” was intended to convey the unorthodox and the unique nature of our approach and not to convey the new introduction of REDCRAFT. We have modified the narratives to clarify this distinction. We have also included a discussion related to the new features of REDCRAFT including the Adaptive Decimation that plays an important role in structure calculation by this software.

Comment 1.12: On lines 116-117, the authors state the novelty of their work. Please make it easier to find! Do not bury the lede!

Response 1.12: We have revised the REDCRAFT section substantially to accomplish multiple objectives. First, we have made sure that the history of REDCRAFT has been clearly presented. We have also included a section highlighting the new features of REDCRAFT with a focus on the Adaptive Decimation. Furthermore, we have better exposed the main points of this manuscript. We also provide a succinct summary of what is new in the current work compared to previous methods, and compared to our previous description of REDCRAFT

Below is an example of our modifications:

In addition, the newest version of REDCRAFT integrates existing dihedral information either from experimental sources such as TALOS^78^, or knowledge bases such as PDB^37^ or PDBMine^79^. In this report, we present the Adaptive Decimation feature that has enabled the use of less RDC data to determine 3D structures of larger proteins. We will begin our discussion of REDCRAFT with some of the key features that enable unique analysis of RDCs and extend the discussion to introduce Adaptive Decimation.

Comment 1.13: On line 134, the authors make the assumption that because you can collect some RDCs for a (large) protein, you could collect more and thus pave the way to a RDC-only structure. I am not an expert in RDCs, but I would be very surprised if this is the case. Often, you can only get a few NOEs for a system.

Response 1.13: We have modified this statement to better convey our point. One of the challenging steps in acquisition of RDCs is the successful preparation of good quality sample and identification of one or more compatible alignment media. Therefore, the successful acquisition of ^15^N-^1^H RDC data is a good indication that other RDC data types can also be obtained.

Comment 1.14: On line 145 the authors state that they calculate RMSDs as a range, when there are missing fragments of the protein. Why not simply present the RMSD over the existing atoms?

Response 1.14: We have refined this segment of the narratives to better explain our approach. In short, due to existence of data gaps, we have conducted structure determination in segments and therefore have one BB-RMSD per segment. We have explained this concept in several places including pages 4-5, 8-9, and 13-14. Below is an example of this clarification:

REDCRAFT generates backbone conformations of proteins from backbone RDCs that is continuous along the protein sequence. Segments of residues for which RDC data is not available, or not properly interpreted because of internal dynamics, result in fragmentation of the available RDC data. In these cases, REDCRAFT supports structure determination of the backbone structure fragments where RDC data are contiguously available.

As explained above, in some cases backbone RDC data are missing for a portion of a protein. In such instance, REDCRAFT accommodates fragmented structure determination and therefore, the structural comparison to the target structure is reported as a range representing the combined BB-RMSD’s calculated for each fragment separately (columns 3 and 4)…

Comment 1.15: When describing the data used for GB1 (lines 155 and below), the authors should state how many RDCs of each class they used in the final set of 209.

Response 1.15: This information is available in Table 4. The late appearance of this table is partially imposed by the required format of the publication venue.

Comment 1.16: On Table 1, the Q factors for some structures are quite high. For example, the REDCRAFT GB3 structure has a range of 0.02 to 0.34, which according to the authors' own definition, corresponds to saying 'extremely good to bad' fit. SR10 has Q factors of ~0.7 and above, so, very poorly fit. Could the authors explain, or maybe illustrate with a figure, which regions are well fitted to the data and which are not?

Response 1.16: We thank the reviewer for bringing this issue to our attention. The reported high Q-factors from the REDCRAFT analysis was based on bookkeeping errors that we have now rectified. The interpretation of Q-factor that we have provided was intended to be used for structure calculation purely from RDC data. The interpretation of Q-factors in the presence of other NMR restraints (and usually far more than RDCs in quantity) will require additional investigations. The proper interpretation can be provided in light of Figure 4. In this figure a Q-factor of ~0.7 corresponds to uncertainty in the BB-RMSD of 3-12 Å. Presence of other NMR restraints can drive and assure structural quality without making an impact on RDC fitness. In fact, this is the case with SR10 where high Q-factors are reported, but structural similarity between NMR and X-ray structures is approximately 3 Å.

Comment 1.17: On the same note, I would find it interesting to see the RMSD of the ensemble of final structures, as a measure of convergence of the structure determination process.

Response 1.17: We have included an ensemble of top 50 structures reported by REDCRAFT for GB1 and PF2048.1 in the supplementary material (S2 and S3 Figures). The top 50 GB1 structures exhibited pairwise BB-RMSD of less than 0.5 Å, while the top 50 structures of PF2048.1 exhibited pairwise BB-RMSD of less than 1.0 Å. The reported BB-RMSDs are based on raw REDCRAFT structures without any refinement.

Comment 1.18: On a more general note, the number of RDCs picked for each case seems quite arbitrary. If I was developing a method like REDCRAFT, I'd like to know what is the lower limit (ballpark) of data I need to use to get a good structure. As the authors state throughout the manuscript, researchers often use 'an excessive' amount of RDCs. It would be interesting to pick one of these cases (or all of them, they are not so many), and take a random sample of 50 RDCs, 100 RDCs, 150 RDCs, ..., and calculate when the structures start to converge to a fold. Maybe there is a relationship to be found between protein size/type and the optimal number of RDCs?

Response 1.18: We fully agree with this comment. We have conducted experiments similar in nature to what the reviewer has suggested using simulated RDC data (see references 90 and 99 in paper). In these experiments, backbone {N-H^N^ and Ca-Ha} provide the most structural information. Our choice of RDCs in this study is governed by two factors. The first is the availability of the RDC data in BMRB. While some investigators may choose to collect Ca-Ha RDCs, others may choose to collect C’-Ca. Therefore, we did not have the flexibility of choosing a common dataset across all proteins. The second step consists of removing the RDC data that cannot be obtained from perdeuterated proteins, which eliminates the inclusion of Ca-Ha. The absence of alpha protons in perdeuterated proteins removes this as a potential candidate in this work.

Comment 1.19: The authors state on lines 205-206 that not using RDCs resulted in a Q factor of ~0.33, but using RDCs lowered it to ~0.28. Why such a small drop? I expected that optimizing the structure using RDCs and then comparing it with respect to how well it fits the data would yield much better Q factors. Can the authors comment on this?

Response 1.19: For this protein target, there are a very high density of NOEs. RDCs cannot significantly improve the structure without violating these NOE-based restraints. Indeed, a Q score of 0.33 is an acceptable score in the presence of other restraints to assure structural quality. The defined evaluation of structural quality based on Q-factor is for strictly RDC-based structure determination in the absence of any other experimental restrains. This point is now made on lines 150-171.

Comment 1.20: On line 220: 20.000 rounds of Powell? To resolve minor van der Waals (typo in the manuscript, please correct with appropriate capitalization) clashes? From experience, 50 steps of Powell are more than enough to clear clashes. Does XPLOR-NIH even get to the 20.000th step or it just converges before?

Response 1.20: We have corrected the spelling error, thank you. In our experience, XPLOR’s minimization does not terminate as quickly as this reviewer’s experience. It is important to note that our minimization rounds must include the RDC force fields, which can add to the complexity of the search space. It is also noteworthy that REDCRAFT will produce the backbone structure of a protein (side chains are not present). These can be the explanation for some of the differences between our observations and the reviewer’s.

Comment 1.21: Related to the structure determination of PF2048.1, a shift from 2.3 to less than 1.0 A with minimization is quite substantial. I would wager that there is a lot that that EM step is doing in terms of optimizing the structure.

Response 1.21: We fully agree with this observation. We have no contention with the impact and importance of final rounds of refinement using any of the traditional software packages. This is not an unexpected change in BB-RMSD and the results are displayed in Figures 2A and 2B. The primary difference is the separation of the two helices (and potentially other helices) that are shown in the red structure (before minimization). This translational shift can easily explain the 1 Å change in structural fitness. The important point that we like to report is REDCRAFT’s ability to determine the structure of PF2048.1 using only backbone {C’-H, N-H^N^, C’-N} to within 2 Å of the actual structure. Inclusion of a refinement step (using the legacy software) allows structure determination to within 1 Å using only the limited set of RDCs. We have promoted final refinements in legacy software at various places in the text. An example is show below:

To demonstrate the capabilities of REDCRAFT, our reported structures (except PF2048.1) have not been subjected to any structural refinement. While we have refrained from refinement steps in this work, clearly all structures can benefit from additional refinement using RDCs and any other experimental data.

Comment 1.22: On line 269: 'as little as 11% of the previously used data' is cherry-picking the lowest value. The average reduction is closer to 25%.

Response 1.22: We modified the text to simply report the range of reductions instead of any other metric. The only outlier in the results is the Rubredoxin with the reduction of 40%. It is important to note that Rubredoxin was previously folded using a predecessor of REDCRAFT and therefore it did not exhibit as much data reduction as other proteins. The improved reduction is due to agree that in the absence of the results shown in Table 1, 11% would have not portrayed a full picture. Our statement is to highlight the extreme potential. We have modified this section to provide a less biases description of the results.

Comment 1.23: The section on RDCs is extremely length and unnecessarily detailed. Please summarize it to the bare minimum to understand the methods used in the manuscript and refer readers to reviews on the topic for more in-depth descriptions.

Response 1.23: We have substantially reduced this section. It now points to the previous work and states the needed topics for this manuscript.

Comment 1.24: On lines 346-347 the authors state that the energy landscape of RDC data is too complex to be navigated by GD/MC methods. However, if we refer to their own example of PF2048.1, it seems that simple EM was able to refine that structure quite substantially, using RDC data. I agree with the authors that specialized algorithms to handle RDC data are necessary and could probably be better than those written in the 80s (CNS/XPLOR), but the data presented in the manuscript does not back this statement.

Response1.24: We have come to realize that a convincing discussion of energy landscape will require additional material such as the figure below. Addition of such material with the needed explanation may not be appropriate at this point. We have motivated the same concept by using Figure 1. Regarding the point made by this reviewer regarding PF2048.1, we respectfully disagree with this statement. The success of “EM” in improving the structure from 2 Å to 1 Å should not be mistaken for a general success in folding of the protein from RDCs. Structure refinement from 2 Å to 1 Å is an entirely different task than folding a protein from 40 Å or more away from the native structure. The inability of “EM” in folding of a protein from only RDCs has been reported by many investigators other than us.

Figure 1. Conformation RDC-energy landscape of 10-th residue (dihedrals of residues 1-9 fixed to the correct angles) in a 10-residue peptide. Note the number of local minima.

Comment 1.25: On line 352, the authors again refer to developments in 'recent years' and refer to REDCRAFT. Again, 2008 is 12 years ago, not necessarily recent. Please rephrase.

Response1.25: We have modified the manuscript (including this sentence) to better address the timeline. As it was mentioned in response to other similar comments, the term recent is relative to the history of NMR spectroscopy that has consisted of more than 70 years of development.

Comment 1.26: Line 354: remove novel from the description of the search algorithm. The current manuscript does not describe any new methodology or feature, so I assume the two-stage method is the same as the original REDCRAFT paper, again, of 2008.

Response 1.26: As recommended, we have refrain from using the term “novel.” Similar to previous comments, our intention in the use of ‘novel’ was to reflect its uniqueness.

Comment 1.27: On lines 369 and onwards, the authors describe how REDCRAFT is the only OOP software available to date. I hope they mean in the field of structural biology. OOP is only useful for developers and is quite tricky to get right. The authors rightfully state that one of the strengths of the paradigm is the ease of adding extensions to the original code. However, the examples they give (Polypeptide built from Atom and AminoAcid objects) are not novel nor unique to REDCRAFT. Biopython's PDB parser, written in 2003, has a similar hierarchical structure for its Structure representation (incidentally also using OOP). Without a very well-documented description of the API, the code base, the classes, their methods, it's nearly impossible for anyone to 'easily' extend REDCRAFT. Indeed, I see the authors have on their webpage a nice readthedocs page, but there is no description of the code/API. See the OpenMM documentation for a very good example of what you should aim for if you want to attract developers to extend your project.

Response1.27: We acknowledge the importance of API in further expansion of the software by the community of developers. However, operating under limited resources, we are currently aiming to establish the usefulness of REDCRAFT before embarking on the development of a full set of documents. Regarding the existence of other OOP platforms, we have modified the text according to the reviewer’s recommendation. The comparison of BioPython’s versus REDCRAFT’s approach to OO encapsulation is one that may not be appropriate in this venue. In short, REDCRAFT’s implementation of Atom class will include biophysical properties of atoms such as (not all have been implemented yet) charges, VDW radius, valance, and other atomic attributes. Amino acids are capable of performing internal rotation of atomic coordinates based on alteration of dihedral angles including the omega angle. PolyPeptides can be created from simple creation and elongation of residues. This approach has many advantages from programmatic perspective. In short, both use OOP but they aim to encapsulate different components for different approaches.

# **Reviewer 2**

The manuscript by Cole, Daigham, Liu, Montelione and Valafar talks about solving protein

structures based on residual dipolar couplings (RDCs) alone, with no recourse to traditional NOE

data. This appears to be an attractive route, given that collection and analyses of NOEs is the bottleneck in the conventional structure-solving routine. The authors present a small set of structures that have been solved by means of their program REDCRAFT using RDCs alone. The set includes a *de* *novo* structure of small archaeal protein PF2048.1, which has no sequence homologues in the PDB. The ability of REDCRAFT to solve structures is truly impressive (especially given an early skepticism in the field). It would definitely be useful to make bio-NMR community aware of these remarkable capabilities.

At the same time, as argued below, there is probably a good reason why people do not solve

structures based on RDCs alone, but rather prefer to rely on NOEs (and/or other data such as

chemical shifts, PREs, etc.). It should be pointed out that the manuscript is unusual in that it reads a bit like an advertisement of REDCRAFT (which is not a new software and has been quite

extensively covered by the authors before). On balance, I suggest that the paper should be published – after revising some of the more general aspects of the discussion + addressing a number technical glitches.

General Response: We thank this reviewer for the recognition of our accomplishment and their constructive feedback. We fully agree with all the points that have been raised by this reviewer regarding the use of RDCs. In fact, our work is motivated by the noted concerns and we feel that we have a theoretically sound solution. We have made sure to follow the recommended changes by this reviewer and have modified the narratives to better address some of the questions. We are certain the modifications resulted from these comments have significantly improved the readability and the scientific content of our manuscript. Thank you!

In particular, we agree that in most cases, one would ideally combine RDC data with chemical shift, NOE, and other available experimental data. Indeed, we have demonstrated the utility of dihedral restraints (such as TALOS) in some of our previous work. The demonstration that backbone structures can be modeled from RDC data alone is provided as proof of concept, rather than a recommended strategy. This point is now made clearly at numerous places such as the example shown below:

To demonstrate the capabilities of REDCRAFT, our reported structures (except PF2048.1) have not been subjected to any structural refinement. While we have refrained from refinement steps in this work, clearly all structures can benefit from additional refinement using RDCs and any other experimental data.

Comment 2.1: I would argue that the advantage of RDC route over the more traditional routes is largely fictional. Indeed, one needs to make 2-3 aligned samples. This is a challenge – for a given protein, some alignment media would work, while others would not (the sample would crash out, etc.). This means 4-6 sample preps, only half of which typically proves to be successful. This also means that the lab should be equipped with the materials and tools to make such multiple aligned samples. After that one needs to record N-HN RDCs (which is not difficult), but additionally some longer-range RDCs, which normally requires several 3D experiments. Clearly, we are talking here about the investment of minimum 1 month, and more likely several months in the case of an average graduate student. On the other hand, NOESY experiment does not require any samples beyond the one that already has been used for resonance assignment, the measurement itself takes several days to a week, while data analysis is largely automated nowadays and does not take much time either. So why should one choose the RDC-based route?

Response 2.2: We fully agree that the collection of RDCs will impose additional steps that may not be the most advantageous path to the structure of all proteins. In some cases – extensive NOESY data can be obtained, and the RDC data is most valuable for improving the accuracy of the models (as shown in Figure 4 of our manuscript). In other cases, only incomplete or sparse NOESY is available (e.g. for perdeuterated proteins), and RDC data is critical for driving the structure modeling. In these studies, we provide proof-of-principle of generating structures from RDC data alone as part of an analysis pipeline that includes the use of legacy software and other NMR data. A second contributing factor to poor utilization of RDCs relates to the complexity of their analysis as noted in comments 2.2 and 2.3. As discussed in response to comments 2.2 and 2.3, REDCRAFT provides solutions to these challenges. We have modified the manuscript to more emphatically state these challenges in several instances. One example is shown in the text below.

RDC data observed from any site on a protein will be influenced by the general alignment (anisotropic tumbling), structure, and the internal dynamics of the protein. Therefore, the proper use of RDCs must include a concurrent treatment of all three aspects of the data, which in turn increases the complexity of their analysis. An incomplete analysis of RDCs can have severe consequences such as generation of an inaccurate structure^35,42,43^. On the other hand, the proper detangling of the three components can provide information about the alignment, structure, and the internal dynamics of the protein on biologically-relevant timescales^73-75^ from a single source of data. In addition to the challenging nature of their analysis, RDCs impose an additional required step of creating a compatible alignment environment. Although RDCs impose this additional sample preparation step, their acquisition may be well justified in some instance when sufficient traditional NOE data are not available (e.g. for perdeuterated proteins).

Comment 2.2: To further elaborate on this point, both RDC and NOE data contain errors due to misassignments, overlaps, etc. The NOE-based structure-solving procedure is very stable with respect to a small number of erroneous data. What about the RDCs? This aspect is not addressed in the paper at all (how to handle the outliers) – and I suspect that the procedure can be derailed by a small number of outliers. What happens when there is a gap of several residues – no RDC data from a stretch of several contiguous residues (or very few RDC data). This would seem to derail the structure-building procedure because we would lack the information about the “translation” of the peptide chain (RDCs only carry the orientational information). The authors should explain this better instead of simply saying that “any existing gaps of less than 6 amino acids can easily be filled during the process of structural refinement”(?) Is it possible to build “mirror structures” given the degeneracies that are present in the RDCs (at least for local fragments, where few RDCs are available because of spectral overlaps, etc.)?

Response 2.2: As a general principle, we agree that the effect of erroneous data is more pronounced when operating with sparse data. However, the effect of noisy RDCs is not as catastrophic as one would imagine. To better address this point, we distinguish between random noisy versus systematic noisy data. We have modified and dedicated a substantial portion of REDCRAFT section to Adaptive Decimation that is designed to mitigate the effect of random noise in the data. We have demonstrated the resilience of REDCRAFT in structure determination with as high and ± 4Hz of uniformly distributed noise (see reference 80 in manuscript). Systematically noisy data such as due to internal dynamics is a far more pathological condition. We have modified the manuscript to include a more extensive discussion of these issues and the capacity of REDCRAFT to resolve these circumstances. Below are some example text that have been incorporated in our revisions:

The presence of noisy RDC data may push the fitness of the globally optimal conformation to beneath the allotted depth search, at which time REDCRAFT will fail to produce the correct structure. The REDCRAFT’s search mechanism has included a Decimation feature to allow representative members (selected based on clustering of structures) of the rejected conformations to proceed to the next round of elongation based on a static cutoff fitness to the RDC data. The Decimation feature is critical in improving the resilience of REDCRAFT to some quantity of erroneous or missing RDC data…

A more effective decimation process will enable recovery from more erroneous or missing RDC data as demonstrated recently^80^.

In this report we demonstrate the application of Adaptive Decimation in structure calculation of proteins as large as 573 residues with a more challenging set of RDC data that can be collected from perdeuterated proteins.

The reviewer is also correct to point out that REDCRAFT will model segments of residues with continuous RDC data. Gaps in the backbone RDC data result in multiple segments. Although RDCs do provide information about the relative orientations between these modeled segments, information about the translation between segments must come modeling restraints, or other data such as NOE or PRE data. This point, also made in Response 1.17, is now discussed at numerous places with an example shown below:

One feature of REDCRAFT is that gaps in RDC data along the backbone result in segments of modeled structures with limited information on how these segments are positioned with respect to each other. In particular, RDC data are insensitive to translational modifications. Under experimental conditions, gaps in the RDC data can be expected, in which case we have demonstrated REDCRAFT’s ability to calculate structural fragments for regions without such data gaps. Although RDC data from two alignment media can be used to orient two rigid domains with respect to each other^47^, they cannot restrain the translational relationship between the two domains. Therefore, restrained energy minimization of the fragments resulting from REDCRAFT modeling and/or inclusion of complementary NMR restraints, are critical in finalizing the structure determination of the protein.

Comment 2.3: The effect of dynamics. Per-residue RDCs can be almost arbitrarily scaled by dynamics. As pointed out by the authors, having an extensive set of experimental RDCs (like 10 pieces of data per residue), one can separate “dynamics” from “structure”. How is it done in the current treatment, though? It seems that the local dynamics is ignored in this manuscript – isn’t there a price to be paid in terms of accuracy?

Response 2.3. Reviewer 2 is absolutely correct to point out that per-residue RDCs will be affected by internal dynamics, which will produce inaccurate structures if left untreated. To address this important point, we have modified the manuscript in numerous places (some examples are shown below) to stress this fact. We have also provided references to our previous work that directly addresses REDCRAFT’s treatment of internal dynamics. When efforts are made to model a static structure to NMR data obtained from dynamic regions of the structure, most current methods will result in inaccurate structures. NOESY or chemical shift have the same problem. REDCRAFT provide an alternative solution to this problem (see reference 42 in manuscript). In short, the gradually increasing fragment size employed by REDCRAFT allows for identification of the onset of internal dynamics. REDCRAFT creates “Dynamic Profile” that informs the user of presence of internal dynamics for proper interpretation of the results. If the dynamical domain of the protein undergoes rigid-body dynamics, REDCRAFT can determine its structure and confirm the existence of internal dynamics by comparing the order tensors obtained from each domain. Using the disparities observed in the order tensors obtained for each domain, a discrete-state model of dynamics can be created with high degree of accuracy. When available, other NMR data (chemical shift anomalies, Lipari-Szabo order parameters, etc.) can always be used to corroborate the findings of REDCRAFT.

REDCRAFT was previously introduced^12^ to provide a more robust means of structure determination and detangling of structural information from internal dynamics^43,76^.

… The gradually increasing problem complexity has several computational and analytical advantages, as well as practical implications. The gradually increasing protein size allows REDCRAFT to avoid the pitfall of starting structure calculations from a fully extended structure (as outlined in Figure 4). The addition of one amino acid at a time allows for identification^42,43^, and characterization of internal dynamics as illustrated elsewhere^42^, therefore removing the effect of dynamics on structure.

Comment 2.4: One should also note that REDCRAFT approach only generates backbone folds (i.e. no side chains). Even though the authors mention that there are computational tools to rebuild side chains, this is a major handicap – the resulting structure is essentially a partial model.

Response 2.4: Agreed. In some cases, backbone structure is sufficient to address key structure /function questions. In other cases, the backbone structure can be used to guide analysis of sidechain assignments and structure from additional NOE or other NMR data. This point is now addressed in the Discussion page 11.

Comment 2.5: The accuracy is also not great. Aside from small model proteins, the accuracy seems to be ca. 2 Å. This is, of course, not suitable for any precision work (such as rational drug design) – especially in the absence of experimentally determined side-chain coordinates as mentioned above. On the other hand, to be fair, the accuracy of conventional NMR structures is also not great (when assessed against X-ray structures) – the authors may want to discuss this aspect as well. The authors say “Although NMR studies may in general be more time consuming and costly than X-ray crystallography”. They should also mention less accurate – this is a major consideration from consumers’ standpoint.

Response 2.5: As noted by the reviewer, the structures produced by REDCRAFT exhibit backbone accuracy relative to X-ray crystal structures and to NOE-based NMR structures. Here, accuracy needs to be defined by some standard reference. In cases where significant differences are observed between NOE-based models and the corresponding X-ray structures, the X-ray structure coordinates are often found to be inconsistent with the NOE data. Hence, the X-ray crystal structure may be very accurate relative to what is present in the crystal, but not accurately reflect the dominant structure in solution.

Comment 2.6: Generally, my feeling is that the authors oversell the advantages of RDCs and of their strategy. For instance, they say: “Indeed, in the 10 ns – 1 s timescale window, RDCs are the most sensitive of NMR parameters(32)”. This cannot be true. While RDCs are the only parameters with some sensitivity to dynamics in the 10 ns – 10 us range, this information is very difficult to get at. There has been a major debate in the field, involving Bax, Clore, Griesinger, Bruschweiler, Torchia, Tolamn and others about getting a handle on dynamics using RDCs – precisely for the reason that this is such a muddy business. In the 10 us – 1 s range there are many NMR experiments that provide a much more clear-cut answers on dynamics. It would be really helpful if the authors present a more balanced view of their RDC-based method instead of a sales-pitch narration.

Response 2.6: We agree that the capability of RDCs in recovery of dynamics is an evolving topic. In this manuscript, we would like to avoid the discussion of dynamics due to the complexity of the topic. Here we only aim to report the ability of REDCRAFT in structure determination of static structures. We do acknowledge the effect of dynamics on RDCs and direct the readers to our previous work that elaborates on the ability of REDCRAFT to properly treat dynamics. Reference to our work have been included in this revision (example shown below).

.. The addition of one amino acid at a time allows for identification^42,43^, and characterization of internal dynamics as illustrated elsewhere^42^, therefore removing the effect of dynamics on structure. …

Comment 2.7: The authors also seem to be wedded to the idea of using RDCs alone. They occasionally use XPLOR-NIH for structure refinement, which actually makes a big difference (see Tab. 2). But they seek to downplay it, e.g. “Aside from completion of the missing residues, these minimizations normally resulted in structural variation of less than 0.5A”. Why insist on using RDCs alone? It would be a good idea to exploit force fields (physics-based or knowledge-based such as ROSETTA). It would be a good idea to throw in other NMR data such as chemical shifts – those are free, they do not require any special measurements (since the assignment is needed anyways). The authors say: “[Certain studies] resorted to the use of other experimental data such as NOEs, dihedral restraints and hydrogen bond restraints (21, 65, 66) for successful structure determination. Both of these approaches nullify the advantages of using RDCs (minimal data, reduced cost)”. This is, generally speaking, not true. Dihedral angles derived from chemical shifts (TALOS) do not cost anything extra. PREs cost relatively little and are quite informative. State of the art force fields are cost-free. At the same time, as we have discussed above, RDCs are actually rather costly. As a methodological point it is interesting to demonstrate that structures (or rather backbone folds) can be successfully obtained by using RDCs alone. But the way into the future is to (optimally) combine RDCs with other pieces of experimental data and with computer modeling.

Response 2.7: As explained in our general response to Reviewer 2 above, we agree that in most cases, one would ideally combine RDC data with chemical shift, NOE, and other experimental data. Indeed, the revised version of the manuscript indicates the ability of REDCRAFT to incorporate dihedral restraints derived from chemical shift data (shown below). The demonstration that backbone structures can be modeled from RDC data alone is provided as proof of concept, rather than a recommended strategy. This point is now made clearly in the Discussion and on line 105 of the revised manuscript. Finally, our statement regarding the use of legacy software was not intended to downplay their important role in structure determination; it was originated from the obvious role they play in the realm of structure determination. We have highlighted and promoted the use of legacy software at numerous places including the Discussion section and the Results section. Below is an example of such modification.

… In addition, the newest version of REDCRAFT integrates existing dihedral information either from experimental sources such as TALOS^78^, or knowledge bases such as PDB^37^ or PDBMine^79^. …

To demonstrate the capabilities of REDCRAFT, our reported structures (except PF2048.1) have not been subjected to any structural refinement. While we have refrained from refinement steps in this work, clearly all structures can benefit from additional refinement using RDCs and any other experimental data.

Comment 2.8: The manuscript is structured in a bit of an awkward way. Over the first 23 pages it talks about the unique advantages of REDCRAFT without actually saying what’s the origin of these advantages. The material at p. 23, which talks about REDCRAFT algorithm, rather belongs to an introduction. Also it needs to be extended and qualified to describe all the limitations mentioned above. In the Materials & Methods, the authors say “In this report we will demonstrate the success of REDCRAFT”, but that’s actually near the end of the paper.

Response 2.8: We have revised the manuscript extensively especially the RDC and REDCRAFT sections. The REDCRAFT section now starts on page 12 and directly focuses on the functional advantages (differences) of REDCRAFT starting on page 14 (line 447). The formatting imposed by the publication venue requires the presentation of Results prior to the Methods section.

Comment 2.9: In some ways, Fig. 5 demonstrates the advantages of NOEs over RDCs – not the other way around. Definitely much better radius of convergence. Also the authors need to add a panel to this graph, zooming in on the range 0-0.05 along the y-axis. Only with this zoom-in we can form an impression about the ability of NOEs to guide structure-optimization procedure toward the global minimum. The existing plot is misleading in this sense simply because of the plotting scale.

Response 2.9: The requested magnified region has been added to this figure (now Figure 4) and is included below. We have added a discussion of this figure for proper interpretation (shown below). This figure clearly illustrates the following points: complementarity of NOEs and RDCs, insensitivity of RDCs when far from the native structure, and insensitivity of NOEs as the search approaches the native structure. The flattened property of the NOE sensitivity is not a plotting anomaly as both sets are plotted in a normalized scale.

… we present Figure 4 that highlights the strengths and weaknesses of structure determination by RDCs and NOEs. Figure 4 represents the RDC and NOE fitness of 5000 derivative structures for PDB-ID 2KDI and 1GB1 (plotted on y-axis) as a function of their BB-RMSD to the known structure (shown on the x-axis). These 5000 structures have been derived from the native structures by randomly altering the backbone torsion angles to achieve a continuum of distortions (measured in BB-RMSD’s). The normalized fitness to both NOE and RDC experimental data (similar to Q-factor^31^) are calculated and plotted on the vertical axis. This figure illustrates the complementarity of NOEs and RDCs as reporters of protein structures. This figure suggests that NOEs are a relatively more sensitive reporters of structural fitness when the search is far from the native state. Therefore, NOEs are relatively more effective in guiding an extended initial structure toward the native structure. However, NOEs lose sensitivity to structural variation as they approach the native structure (as the curve flattens). Therefore, NOEs become relatively insensitive to structural variation when less than 5 Å from the native structure. RDCs on the other hand exhibit a lack of sensitivity to structural variation when far from the native structure but gain sensitivity as they approach the native structure (steep incline and the tight scattering of the curve). The insensitivity of RDCs in structure determination from an extended structure is the primary cause of failure when using an extended structure as the starting point of search. The sensitivity of RDCs is generally the impetus in developing RDC based structure determination approaches. The existence of internal dynamics further complicates their use in structure determination endeavors.

Normalized Fitness Score

| BB-RMSD (Å) | 2kdi-RDC |
| --- | --- |
| 8.94669 | 0.9831 |
| 14.9441 | 0.985159 |
| 12.0621 | 0.950194 |
| 9.84241 | 0.913291 |
| 1.57487 | 0.227887 |
| 8.99155 | 0.888907 |
| 7.9178 | 0.893349 |
| 8.11825 | 0.940332 |
| 8.4736 | 0.912527 |
| 4.67765 | 0.672522 |
| 10.5503 | 0.936828 |
| 9.70294 | 0.965671 |
| 1.60851 | 0.247093 |
| 4.24451 | 0.588668 |
| 2.67735 | 0.313604 |
| 3.54644 | 0.477212 |
| 4.17162 | 0.54643 |
| 13.1316 | 0.955242 |
| 11.7334 | 0.956892 |
| 18.1177 | 0.996458 |
| 14.928 | 0.970794 |
| 10.7402 | 0.903089 |
| 10.7164 | 0.982381 |
| 5.91189 | 0.725465 |
| 3.7581 | 0.592194 |
| 14.1935 | 0.995459 |
| 10.0394 | 0.891866 |
| 11.5217 | 0.985749 |
| 4.33492 | 0.573667 |
| 11.8385 | 0.982495 |
| 12.7209 | 0.965769 |
| 14.8691 | 0.978741 |
| 12.8443 | 0.974102 |
| 11.4313 | 0.995928 |
| 3.79385 | 0.567048 |
| 3.02989 | 0.424383 |
| 9.21583 | 0.949573 |
| 19.9441 | 0.961614 |
| 4.82413 | 0.58239 |
| 19.532 | 0.947235 |
| 4.20736 | 0.566169 |
| 5.16266 | 0.558534 |
| 10.2384 | 0.956195 |
| 9.68051 | 0.973685 |
| 4.9566 | 0.560351 |
| 9.23195 | 0.940983 |
| 9.84381 | 0.899048 |
| 7.66619 | 0.92366 |
| 8.97893 | 0.9268 |
| 12.2702 | 0.992371 |
| 6.10884 | 0.915592 |
| 11.247 | 0.964233 |
| 5.16266 | 0.867405 |
| 10.2006 | 0.971899 |
| 1.70944 | 0.235454 |
| 12.9739 | 0.902771 |
| 11.111 | 0.936889 |
| 2.32831 | 0.329698 |
| 1.77041 | 0.224003 |
| 10.8208 | 0.917651 |
| 11.1495 | 0.956036 |
| 13.4968 | 0.981027 |
| 1.97998 | 0.296039 |
| 9.53963 | 0.95649 |
| 9.20742 | 0.972066 |
| 13.0924 | 0.960403 |
| 10.1094 | 0.896482 |
| 2.45797 | 0.379547 |
| 8.82053 | 0.884245 |
| 13.5767 | 0.957875 |
| 3.9144 | 0.542032 |
| 6.24411 | 0.809304 |
| 12.1182 | 0.995368 |
| 6.33943 | 0.923629 |
| 9.99871 | 0.935458 |
| 12.0018 | 0.996057 |
| 8.99365 | 0.934785 |
| 13.0587 | 0.968312 |
| 2.32831 | 0.511286 |
| 2.91144 | 0.410036 |
| 5.518 | 0.836315 |
| 12.3929 | 0.973625 |
| 8.06288 | 0.937948 |
| 4.16461 | 0.574261 |
| 2.93597 | 0.394759 |
| 9.54734 | 0.959064 |
| 5.97708 | 0.543878 |
| 3.00956 | 0.412285 |
| 7.59119 | 0.914366 |
| 12.3221 | 0.976054 |
| 10.7388 | 0.931758 |
| 18.7786 | 0.942512 |
| 7.58839 | 0.890375 |
| 2.53858 | 0.417359 |
| 11.7775 | 0.962091 |
| 9.86204 | 0.925052 |
| 8.61377 | 0.894424 |
| 8.57032 | 0.841174 |
| 12.8429 | 0.942353 |
| 7.45663 | 0.90844 |
| 12.0221 | 0.921457 |
| 10.7767 | 0.90701 |
| 9.82209 | 0.943155 |
| 14.5979 | 0.989193 |
| 10.4613 | 0.922676 |
| 4.16601 | 0.432545 |
| 13.5907 | 0.960963 |
| 8.97963 | 0.923728 |
| 1.74799 | 0.359399 |
| 10.237 | 0.975335 |
| 13.3538 | 0.973928 |
| 8.0762 | 0.936685 |
| 13.3202 | 0.971127 |
| 10.4382 | 0.848878 |
| 9.89568 | 0.950542 |
| 12.0908 | 0.972157 |
| 13.672 | 0.966276 |
| 16.2323 | 0.956687 |
| 9.01397 | 0.911861 |
| 4.46669 | 0.72805 |
| 1.85732 | 0.255858 |
| 3.63264 | 0.427996 |
| 13.6496 | 0.982132 |
| 1.79565 | 0.269074 |
| 1.71434 | 0.231055 |
| 11.111 | 0.939893 |
| 4.2347 | 0.798951 |
| 8.93407 | 0.984258 |
| 2.7201 | 0.433258 |
| 15.148 | 0.95531 |
| 13.0223 | 0.936261 |
| 2.14889 | 0.501097 |
| 20.5756 | 0.989329 |
| 12.0859 | 0.953312 |
| 11.8602 | 0.949028 |
| 8.94809 | 0.931841 |
| 4.6398 | 0.775293 |
| 4.88791 | 0.549494 |
| 7.4356 | 0.783315 |
| 13.2676 | 0.978363 |
| 1.3611 | 0.282191 |
| 12.8583 | 0.98005 |
| 3.41467 | 0.469653 |
| 6.09342 | 0.780977 |
| 6.93167 | 0.834408 |
| 9.9146 | 0.958746 |
| 8.14489 | 0.847183 |
| 11.91 | 0.984841 |
| 7.37532 | 0.811862 |
| 8.99365 | 0.963317 |
| 12.0936 | 0.957535 |
| 11.6346 | 0.984796 |
| 6.84476 | 0.839395 |
| 12.7188 | 0.94865 |
| 5.69392 | 0.748557 |
| 16.4061 | 0.958867 |
| 11.6549 | 0.954228 |
| 14.3533 | 0.910211 |
| 4.46248 | 0.556505 |
| 8.26824 | 0.887567 |
| 7.20641 | 0.875609 |
| 9.37352 | 0.93007 |
| 7.48326 | 0.938433 |
| 2.7944 | 0.482628 |
| 13.7281 | 0.942822 |
| 4.70779 | 0.655398 |
| 12.1273 | 0.986339 |
| 11.3871 | 0.965489 |
| 9.00907 | 0.899555 |
| 12.0936 | 0.984614 |
| 3.36491 | 0.604916 |
| 14.5032 | 0.957353 |
| 11.214 | 0.956438 |
| 11.7074 | 0.98413 |
| 13.8598 | 0.980459 |
| 12.3663 | 0.973398 |
| 8.65302 | 0.970514 |
| 9.98048 | 0.920693 |
| 3.87865 | 0.528027 |
| 5.36942 | 0.722557 |
| 11.083 | 0.949195 |
| 10.8776 | 0.916046 |
| 10.0541 | 0.933075 |
| 10.3043 | 0.954894 |
| 13.5788 | 0.966004 |
| 7.41457 | 0.76357 |
| 12.5268 | 0.945615 |
| 11.65 | 0.964187 |
| 10.5945 | 0.914199 |
| 10.5356 | 0.945486 |
| 6.77748 | 0.784609 |
| 12.6754 | 0.933332 |
| 2.01993 | 0.282974 |
| 10.3744 | 0.950489 |
| 9.78774 | 0.986476 |
| 3.0411 | 0.446122 |
| 2.29467 | 0.4247 |
| 9.94264 | 0.965913 |
| 4.69587 | 0.658265 |
| 9.87255 | 0.980141 |

| BB-RMSD (Å) | 1gb1-RDC |
| --- | --- |
| 8.464 | 0.893342 |
| 13.873 | 0.943615 |
| 16.062 | 0.895141 |
| 11.556 | 0.905084 |
| 10.973 | 0.955638 |
| 9.669 | 0.91326 |
| 1.324 | 0.15414 |
| 8.226 | 0.840099 |
| 5.247 | 0.549075 |
| 2.309 | 0.240548 |
| 9.362 | 0.952997 |
| 8.641 | 0.917399 |
| 1.495 | 0.171619 |
| 7.831 | 0.804989 |
| 6.943 | 0.694283 |
| 6.241 | 0.747089 |
| 6.654 | 0.712063 |
| 2.199 | 0.168097 |
| 8.353 | 0.938587 |
| 3.477 | 0.611444 |
| 5.106 | 0.729724 |
| 10.426 | 0.875512 |
| 3.169 | 0.492069 |
| 9.992 | 0.946643 |
| 11.332 | 0.989801 |
| 8.489 | 0.942335 |
| 1.572 | 0.213961 |
| 11.592 | 0.917403 |
| 4.585 | 0.521714 |
| 10.556 | 0.939229 |
| 1.69 | 0.239629 |
| 17.862 | 0.929671 |
| 10.433 | 0.959778 |
| 14.491 | 0.964972 |
| 12.397 | 0.956684 |
| 7.199 | 0.872586 |
| 11.968 | 0.900165 |
| 3.813 | 0.697178 |
| 1.75 | 0.303464 |
| 6.715 | 0.779556 |
| 11.725 | 0.892415 |
| 6.903 | 0.528833 |
| 6.02 | 0.631486 |
| 1.35 | 0.188218 |
| 15.122 | 0.886269 |
| 4.867 | 0.704524 |
| 21.159 | 0.925013 |
| 8.866 | 0.806271 |
| 7.834 | 0.873398 |
| 11.03 | 0.936214 |
| 3.53 | 0.368446 |
| 4.536 | 0.57118 |
| 6.144 | 0.926783 |
| 14.581 | 0.901356 |
| 14.703 | 0.941547 |
| 13.098 | 0.851223 |
| 16.326 | 0.900007 |
| 0.92 | 0.0899621 |
| 7.054 | 0.795688 |
| 11.223 | 0.808337 |
| 11.296 | 0.824834 |
| 3.672 | 0.572586 |
| 4.692 | 0.708955 |
| 7.871 | 0.85507 |
| 13.195 | 0.954515 |
| 13.779 | 0.889065 |
| 10.395 | 0.757814 |
| 10.535 | 0.873575 |
| 1.223 | 0.164361 |
| 15.406 | 0.948615 |
| 1.372 | 0.158931 |
| 12.184 | 0.850523 |
| 11.266 | 0.864942 |
| 8.295 | 0.906755 |
| 5.412 | 0.657043 |
| 9.275 | 0.809911 |
| 3.281 | 0.508206 |
| 1.136 | 0.157492 |
| 1.32 | 0.18269 |
| 9.893 | 0.837151 |
| 6.394 | 0.463993 |
| 12.21 | 0.895092 |
| 1.771 | 0.153846 |
| 14.878 | 0.891117 |
| 4.957 | 0.547763 |
| 5.946 | 0.835829 |
| 12.711 | 0.965503 |
| 20.878 | 0.902922 |
| 8.375 | 0.839851 |
| 4.372 | 0.407548 |
| 10.072 | 0.915684 |
| 3.201 | 0.406285 |
| 1.543 | 0.146656 |
| 6.125 | 0.530525 |
| 1.074 | 0.12923 |
| 11.338 | 0.861625 |
| 9.749 | 0.92702 |
| 13.006 | 0.975705 |
| 6.625 | 0.866293 |
| 6.488 | 0.834238 |
| 9.707 | 0.799153 |
| 9.845 | 0.959804 |
| 12.426 | 0.850205 |
| 8.635 | 0.937486 |
| 13.213 | 0.932038 |
| 5.585 | 0.582702 |
| 19.054 | 0.947856 |
| 10.005 | 0.810734 |
| 5.734 | 0.6568 |
| 12.755 | 0.860007 |
| 2.104 | 0.281852 |
| 10.96 | 0.959103 |
| 4.874 | 0.423962 |
| 9.233 | 0.931764 |
| 10.677 | 0.919299 |
| 2.589 | 0.364508 |
| 2.369 | 0.221362 |
| 15.519 | 0.945914 |
| 4.792 | 0.639064 |
| 21.031 | 0.995559 |
| 13.093 | 0.88602 |
| 0.991 | 0.108249 |
| 11.435 | 0.815079 |
| 5.436 | 0.86028 |
| 13.096 | 0.920395 |
| 4.166 | 0.620125 |
| 1.111 | 0.12178 |
| 13.659 | 0.963098 |
| 11.915 | 0.759388 |
| 10.528 | 0.936297 |
| 7.602 | 0.912184 |
| 7.718 | 0.861252 |
| 15.537 | 0.847951 |
| 8.906 | 0.864349 |
| 6.459 | 0.887803 |
| 4.971 | 0.746717 |
| 11.043 | 0.897002 |
| 9.177 | 0.75103 |
| 10.549 | 0.710632 |
| 8.202 | 0.910916 |
| 3.031 | 0.443781 |
| 7.024 | 0.605998 |
| 7.276 | 0.867282 |
| 1.057 | 0.129258 |
| 8.18 | 0.833907 |
| 1.735 | 0.18232 |
| 9.906 | 0.956756 |
| 6.578 | 0.645533 |
| 15.683 | 0.819277 |
| 2.356 | 0.228969 |
| 4.216 | 0.565738 |
| 10.587 | 0.945747 |
| 7.465 | 0.84881 |
| 9.388 | 0.899644 |
| 14.029 | 0.973283 |
| 1.551 | 0.125946 |
| 10.132 | 0.960028 |
| 9.392 | 0.952153 |
| 3.266 | 0.377406 |
| 11.805 | 0.956782 |
| 11.345 | 0.977321 |
| 10.307 | 0.917564 |
| 11.188 | 0.944502 |
| 2.077 | 0.460653 |
| 14.885 | 0.929037 |
| 6.697 | 0.844201 |
| 2.234 | 0.568883 |
| 1.559 | 0.187117 |
| 17.427 | 0.938895 |
| 9.46 | 0.88647 |
| 1.739 | 0.164756 |
| 14.362 | 0.918381 |
| 11.586 | 0.788373 |
| 8.142 | 0.841199 |
| 10.251 | 0.833989 |
| 4.947 | 0.872049 |
| 11.77 | 0.896601 |
| 19.548 | 0.942964 |
| 6.897 | 0.553768 |
| 1.377 | 0.147678 |
| 15.93 | 0.851151 |
| 2.036 | 0.28668 |
| 7.444 | 0.95288 |
| 13.441 | 0.899841 |
| 8.889 | 0.931441 |
| 12.21 | 0.94885 |
| 1.461 | 0.169154 |
| 12.501 | 0.891952 |
| 11.852 | 0.932728 |
| 7.391 | 0.770633 |
| 9.064 | 0.970925 |
| 5.294 | 0.746964 |
| 4.298 | 0.548231 |
| 4.066 | 0.621436 |
| 8.348 | 0.837298 |
| 10.993 | 0.837346 |
| 10.347 | 0.894532 |
| 11.446 | 0.86486 |
| 3.105 | 0.596196 |
| 2.874 | 0.500654 |

| BB-RMSD (Å) | 2kdi-NOE |
| --- | --- |
| 8.94669 | 0.104963 |
| 14.9441 | 0.248285 |
| 12.0621 | 0.128458 |
| 9.84241 | 0.0683861 |
| 1.57487 | 0.00438513 |
| 8.99155 | 0.0841135 |
| 7.9178 | 0.0344604 |
| 8.11825 | 0.085719 |
| 8.4736 | 0.0627966 |
| 4.67765 | 0.0379936 |
| 10.5503 | 0.100424 |
| 9.70294 | 0.0806697 |
| 1.60851 | 0.00400155 |
| 4.24451 | 0.0110824 |
| 2.67735 | 0.00900736 |
| 3.54644 | 0.013969 |
| 4.17162 | 0.0169197 |
| 13.1316 | 0.205098 |
| 11.7334 | 0.259516 |
| 18.1177 | 0.449349 |
| 14.928 | 0.246963 |
| 10.7402 | 0.0924791 |
| 10.7164 | 0.140313 |
| 5.91189 | 0.0592321 |
| 3.7581 | 0.00874085 |
| 14.1935 | 0.222843 |
| 10.0394 | 0.0519694 |
| 11.5217 | 0.134869 |
| 4.33492 | 0.014457 |
| 11.8385 | 0.197393 |
| 12.7209 | 0.165486 |
| 14.8691 | 0.295249 |
| 12.8443 | 0.190237 |
| 11.4313 | 0.118987 |
| 3.79385 | 0.0194574 |
| 3.02989 | 0.00987263 |
| 9.21583 | 0.0857655 |
| 19.9441 | 0.680017 |
| 4.82413 | 0.0334953 |
| 19.532 | 0.546966 |
| 4.20736 | 0.020537 |
| 5.16266 | 0.0252213 |
| 10.2384 | 0.189522 |
| 9.68051 | 0.0970389 |
| 4.9566 | 0.0222323 |
| 9.23195 | 0.0742787 |
| 9.84381 | 0.122238 |
| 7.66619 | 0.073001 |
| 8.97893 | 0.0862254 |
| 12.2702 | 0.265794 |
| 6.10884 | 0.0302696 |
| 11.247 | 0.0690988 |
| 5.16266 | 0.0169067 |
| 10.2006 | 0.0813295 |
| 1.70944 | 0.00364014 |
| 12.9739 | 0.131942 |
| 11.111 | 0.0982426 |
| 2.32831 | 0.00621761 |
| 1.77041 | 0.00420752 |
| 10.8208 | 0.101995 |
| 11.1495 | 0.088495 |
| 13.4968 | 0.276804 |
| 1.97998 | 0.00596052 |
| 9.53963 | 0.204266 |
| 9.20742 | 0.127667 |
| 13.0924 | 0.243242 |
| 10.1094 | 0.109492 |
| 2.45797 | 0.00446021 |
| 8.82053 | 0.0749928 |
| 13.5767 | 0.226936 |
| 3.9144 | 0.011392 |
| 6.24411 | 0.0396026 |
| 12.1182 | 0.150068 |
| 6.33943 | 0.0567341 |
| 9.99871 | 0.176345 |
| 12.0018 | 0.233585 |
| 8.99365 | 0.129206 |
| 13.0587 | 0.229115 |
| 2.32831 | 0.00475653 |
| 2.91144 | 0.00473476 |
| 5.518 | 0.0341429 |
| 12.3929 | 0.16036 |
| 8.06288 | 0.0474473 |
| 4.16461 | 0.0220398 |
| 2.93597 | 0.0057215 |
| 9.54734 | 0.151522 |
| 5.97708 | 0.0263983 |
| 3.00956 | 0.0151905 |
| 7.59119 | 0.0418945 |
| 12.3221 | 0.293083 |
| 10.7388 | 0.112713 |
| 18.7786 | 0.695737 |
| 7.58839 | 0.0440838 |
| 2.53858 | 0.00791682 |
| 11.7775 | 0.128585 |
| 9.86204 | 0.113942 |
| 8.61377 | 0.149991 |
| 8.57032 | 0.0662707 |
| 12.8429 | 0.221181 |
| 7.45663 | 0.023531 |
| 12.0221 | 0.204512 |
| 10.7767 | 0.133284 |
| 9.82209 | 0.0530463 |
| 14.5979 | 0.335605 |
| 10.4613 | 0.138694 |
| 4.16601 | 0.0161321 |
| 13.5907 | 0.273558 |
| 8.97963 | 0.06958 |
| 1.74799 | 0.00527369 |
| 10.237 | 0.0844007 |
| 13.3538 | 0.273618 |
| 8.0762 | 0.0889724 |
| 13.3202 | 0.17584 |
| 10.4382 | 0.0458781 |
| 9.89568 | 0.0660999 |
| 12.0908 | 0.215098 |
| 13.672 | 0.392952 |
| 16.2323 | 0.323447 |
| 9.01397 | 0.0855523 |
| 4.46669 | 0.0227483 |
| 1.85732 | 0.0045572 |
| 3.63264 | 0.0120704 |
| 13.6496 | 0.314421 |
| 1.79565 | 0.00568763 |
| 1.71434 | 0.00433922 |
| 11.111 | 0.129928 |
| 4.2347 | 0.0152892 |
| 8.93407 | 0.11659 |
| 2.7201 | 0.011532 |
| 15.148 | 0.367564 |
| 13.0223 | 0.209174 |
| 2.14889 | 0.00712255 |
| 20.5756 | 0.788881 |
| 12.0859 | 0.217612 |
| 11.8602 | 0.166496 |
| 8.94809 | 0.0824506 |
| 4.6398 | 0.02434 |
| 4.88791 | 0.0194264 |
| 7.4356 | 0.0720161 |
| 13.2676 | 0.304004 |
| 1.3611 | 0.00292675 |
| 12.8583 | 0.232912 |
| 3.41467 | 0.00630149 |
| 6.09342 | 0.0319849 |
| 6.93167 | 0.0421911 |
| 9.9146 | 0.1472 |
| 8.14489 | 0.0793667 |
| 11.91 | 0.0917473 |
| 7.37532 | 0.0890264 |
| 8.99365 | 0.0586679 |
| 12.0936 | 0.133522 |
| 11.6346 | 0.153755 |
| 6.84476 | 0.0698368 |
| 12.7188 | 0.254258 |
| 5.69392 | 0.0638665 |
| 16.4061 | 0.540889 |
| 11.6549 | 0.216575 |
| 14.3533 | 0.207504 |
| 4.46248 | 0.0248892 |
| 8.26824 | 0.0880835 |
| 7.20641 | 0.0381561 |
| 9.37352 | 0.0761988 |
| 7.48326 | 0.0311279 |
| 2.7944 | 0.00954993 |
| 13.7281 | 0.291807 |
| 4.70779 | 0.0214577 |
| 12.1273 | 0.178008 |
| 11.3871 | 0.244814 |
| 9.00907 | 0.109031 |
| 12.0936 | 0.149508 |
| 3.36491 | 0.00854707 |
| 14.5032 | 0.249508 |
| 11.214 | 0.254626 |
| 11.7074 | 0.198251 |
| 13.8598 | 0.121884 |
| 12.3663 | 0.177816 |
| 8.65302 | 0.145311 |
| 9.98048 | 0.0858207 |
| 3.87865 | 0.0184056 |
| 5.36942 | 0.0273791 |
| 11.083 | 0.0963963 |
| 10.8776 | 0.106561 |
| 10.0541 | 0.120679 |
| 10.3043 | 0.0928291 |
| 13.5788 | 0.273844 |
| 7.41457 | 0.0217402 |
| 12.5268 | 0.233602 |
| 11.65 | 0.222027 |
| 10.5945 | 0.192999 |
| 10.5356 | 0.0975665 |
| 6.77748 | 0.0239199 |
| 12.6754 | 0.138997 |
| 2.01993 | 0.004716 |
| 10.3744 | 0.130933 |
| 9.78774 | 0.0624652 |
| 3.0411 | 0.013038 |
| 2.29467 | 0.00625392 |
| 9.94264 | 0.11315 |
| 4.69587 | 0.014961 |
| 9.87255 | 0.0907589 |

| BB-RMSD (Å) | 1gb1-NOE |
| --- | --- |
| 8.464 | 0.0738209 |
| 13.873 | 0.15083 |
| 16.062 | 0.521217 |
| 11.556 | 0.261561 |
| 10.973 | 0.174366 |
| 9.669 | 0.0872082 |
| 1.324 | 0.00182558 |
| 8.226 | 0.0792641 |
| 5.247 | 0.0262969 |
| 2.309 | 0.00528628 |
| 9.362 | 0.156444 |
| 8.641 | 0.11827 |
| 1.495 | 0.00402449 |
| 7.831 | 0.0584909 |
| 6.943 | 0.0794607 |
| 6.241 | 0.0475137 |
| 6.654 | 0.0216236 |
| 2.199 | 0.00254984 |
| 8.353 | 0.065544 |
| 3.477 | 0.0173881 |
| 5.106 | 0.0287857 |
| 10.426 | 0.0551996 |
| 3.169 | 0.0122063 |
| 9.992 | 0.107015 |
| 11.332 | 0.153946 |
| 8.489 | 0.137029 |
| 1.572 | 0.0013296 |
| 11.592 | 0.196168 |
| 4.585 | 0.0336068 |
| 10.556 | 0.162892 |
| 1.69 | 0.000783783 |
| 17.862 | 0.374768 |
| 10.433 | 0.206171 |
| 14.491 | 0.402867 |
| 12.397 | 0.240914 |
| 7.199 | 0.0792876 |
| 11.968 | 0.21558 |
| 3.813 | 0.0134158 |
| 1.75 | 0.0041804 |
| 6.715 | 0.0432728 |
| 11.725 | 0.0991385 |
| 6.903 | 0.0362039 |
| 6.02 | 0.0416683 |
| 1.35 | 0.00167721 |
| 15.122 | 0.276914 |
| 4.867 | 0.0258193 |
| 21.159 | 0.518896 |
| 8.866 | 0.0534128 |
| 7.834 | 0.0353058 |
| 11.03 | 0.0695643 |
| 3.53 | 0.00542268 |
| 4.536 | 0.0189504 |
| 6.144 | 0.02221 |
| 14.581 | 0.309863 |
| 14.703 | 0.323726 |
| 13.098 | 0.232819 |
| 16.326 | 0.431045 |
| 0.92 | 0.00111653 |
| 7.054 | 0.0369718 |
| 11.223 | 0.162767 |
| 11.296 | 0.322313 |
| 3.672 | 0.00714037 |
| 4.692 | 0.031791 |
| 7.871 | 0.0813718 |
| 13.195 | 0.185459 |
| 13.779 | 0.137271 |
| 10.395 | 0.078563 |
| 10.535 | 0.124305 |
| 1.223 | 0.00096271 |
| 15.406 | 0.231005 |
| 1.372 | 0.00173319 |
| 12.184 | 0.254796 |
| 11.266 | 0.102889 |
| 8.295 | 0.0774286 |
| 5.412 | 0.021243 |
| 9.275 | 0.0661163 |
| 3.281 | 0.0080654 |
| 1.136 | 0.000813693 |
| 1.32 | 0.00132883 |
| 9.893 | 0.116079 |
| 6.394 | 0.0562241 |
| 12.21 | 0.146199 |
| 1.771 | 0.0018337 |
| 14.878 | 0.343602 |
| 4.957 | 0.0208134 |
| 5.946 | 0.0421518 |
| 12.711 | 0.105555 |
| 20.878 | 0.45188 |
| 8.375 | 0.0978173 |
| 4.372 | 0.0176765 |
| 10.072 | 0.148421 |
| 3.201 | 0.00427153 |
| 1.543 | 0.00154751 |
| 6.125 | 0.0397812 |
| 1.074 | 0.00123783 |
| 11.338 | 0.170489 |
| 9.749 | 0.180716 |
| 13.006 | 0.19748 |
| 6.625 | 0.0201749 |
| 6.488 | 0.0157727 |
| 9.707 | 0.15803 |
| 9.845 | 0.113785 |
| 12.426 | 0.144971 |
| 8.635 | 0.111668 |
| 13.213 | 0.232559 |
| 5.585 | 0.0356804 |
| 19.054 | 0.547324 |
| 10.005 | 0.0886536 |
| 5.734 | 0.0412232 |
| 12.755 | 0.114133 |
| 2.104 | 0.00328402 |
| 10.96 | 0.143494 |
| 4.874 | 0.010596 |
| 9.233 | 0.095377 |
| 10.677 | 0.0936066 |
| 2.589 | 0.00763828 |
| 2.369 | 0.0122043 |
| 15.519 | 0.315772 |
| 4.792 | 0.0284724 |
| 21.031 | 0.585458 |
| 13.093 | 0.242235 |
| 0.991 | 0.000807624 |
| 11.435 | 0.155338 |
| 5.436 | 0.0364144 |
| 13.096 | 0.196477 |
| 4.166 | 0.0161241 |
| 1.111 | 0.00140357 |
| 13.659 | 0.23217 |
| 11.915 | 0.194653 |
| 10.528 | 0.0864486 |
| 7.602 | 0.05556 |
| 7.718 | 0.0959129 |
| 15.537 | 0.369574 |
| 8.906 | 0.0866981 |
| 6.459 | 0.0268725 |
| 4.971 | 0.00780042 |
| 11.043 | 0.156966 |
| 9.177 | 0.138418 |
| 10.549 | 0.166705 |
| 8.202 | 0.0647505 |
| 3.031 | 0.0143244 |
| 7.024 | 0.040348 |
| 7.276 | 0.035342 |
| 1.057 | 0.00121416 |
| 8.18 | 0.0585015 |
| 1.735 | 0.00255666 |
| 9.906 | 0.0852477 |
| 6.578 | 0.0615711 |
| 15.683 | 0.15237 |
| 2.356 | 0.00674368 |
| 4.216 | 0.023549 |
| 10.587 | 0.212351 |
| 7.465 | 0.038134 |
| 9.388 | 0.118753 |
| 14.029 | 0.319208 |
| 1.551 | 0.00260092 |
| 10.132 | 0.0543817 |
| 9.392 | 0.0643974 |
| 3.266 | 0.00943447 |
| 11.805 | 0.223433 |
| 11.345 | 0.234229 |
| 10.307 | 0.113261 |
| 11.188 | 0.209528 |
| 2.077 | 0.00486926 |
| 14.885 | 0.31654 |
| 6.697 | 0.0403387 |
| 2.234 | 0.00823716 |
| 1.559 | 0.00189436 |
| 17.427 | 0.487295 |
| 9.46 | 0.0752481 |
| 1.739 | 0.00154391 |
| 14.362 | 0.276862 |
| 11.586 | 0.0981883 |
| 8.142 | 0.053601 |
| 10.251 | 0.092912 |
| 4.947 | 0.0364021 |
| 11.77 | 0.196868 |
| 19.548 | 0.631766 |
| 6.897 | 0.0425056 |
| 1.377 | 0.000881627 |
| 15.93 | 0.197882 |
| 2.036 | 0.00155302 |
| 7.444 | 0.0524824 |
| 13.441 | 0.332453 |
| 8.889 | 0.080852 |
| 12.21 | 0.355023 |
| 1.461 | 0.00366182 |
| 12.501 | 0.243181 |
| 11.852 | 0.162346 |
| 7.391 | 0.0434324 |
| 9.064 | 0.195363 |
| 5.294 | 0.0321061 |
| 4.298 | 0.0115269 |
| 4.066 | 0.0196663 |
| 8.348 | 0.056372 |
| 10.993 | 0.109905 |
| 10.347 | 0.0829908 |
| 11.446 | 0.166774 |
| 3.105 | 0.0111073 |
| 2.874 | 0.00369806 |

Normalized Fitness Score

| BB-RMSD (Å) | 2kdi-RDC |
| --- | --- |
| 8.94669 | 0.9831 |
| 14.9441 | 0.985159 |
| 12.0621 | 0.950194 |
| 9.84241 | 0.913291 |
| 1.57487 | 0.227887 |
| 8.99155 | 0.888907 |
| 7.9178 | 0.893349 |
| 8.11825 | 0.940332 |
| 8.4736 | 0.912527 |
| 4.67765 | 0.672522 |
| 10.5503 | 0.936828 |
| 9.70294 | 0.965671 |
| 1.60851 | 0.247093 |
| 4.24451 | 0.588668 |
| 2.67735 | 0.313604 |
| 3.54644 | 0.477212 |
| 4.17162 | 0.54643 |
| 13.1316 | 0.955242 |
| 11.7334 | 0.956892 |
| 18.1177 | 0.996458 |
| 14.928 | 0.970794 |
| 10.7402 | 0.903089 |
| 10.7164 | 0.982381 |
| 5.91189 | 0.725465 |
| 3.7581 | 0.592194 |
| 14.1935 | 0.995459 |
| 10.0394 | 0.891866 |
| 11.5217 | 0.985749 |
| 4.33492 | 0.573667 |
| 11.8385 | 0.982495 |
| 12.7209 | 0.965769 |
| 14.8691 | 0.978741 |
| 12.8443 | 0.974102 |
| 11.4313 | 0.995928 |
| 3.79385 | 0.567048 |
| 3.02989 | 0.424383 |
| 9.21583 | 0.949573 |
| 19.9441 | 0.961614 |
| 4.82413 | 0.58239 |
| 19.532 | 0.947235 |
| 4.20736 | 0.566169 |
| 5.16266 | 0.558534 |
| 10.2384 | 0.956195 |
| 9.68051 | 0.973685 |
| 4.9566 | 0.560351 |
| 9.23195 | 0.940983 |
| 9.84381 | 0.899048 |
| 7.66619 | 0.92366 |
| 8.97893 | 0.9268 |
| 12.2702 | 0.992371 |
| 6.10884 | 0.915592 |
| 11.247 | 0.964233 |
| 5.16266 | 0.867405 |
| 10.2006 | 0.971899 |
| 1.70944 | 0.235454 |
| 12.9739 | 0.902771 |
| 11.111 | 0.936889 |
| 2.32831 | 0.329698 |
| 1.77041 | 0.224003 |
| 10.8208 | 0.917651 |
| 11.1495 | 0.956036 |
| 13.4968 | 0.981027 |
| 1.97998 | 0.296039 |
| 9.53963 | 0.95649 |
| 9.20742 | 0.972066 |
| 13.0924 | 0.960403 |
| 10.1094 | 0.896482 |
| 2.45797 | 0.379547 |
| 8.82053 | 0.884245 |
| 13.5767 | 0.957875 |
| 3.9144 | 0.542032 |
| 6.24411 | 0.809304 |
| 12.1182 | 0.995368 |
| 6.33943 | 0.923629 |
| 9.99871 | 0.935458 |
| 12.0018 | 0.996057 |
| 8.99365 | 0.934785 |
| 13.0587 | 0.968312 |
| 2.32831 | 0.511286 |
| 2.91144 | 0.410036 |
| 5.518 | 0.836315 |
| 12.3929 | 0.973625 |
| 8.06288 | 0.937948 |
| 4.16461 | 0.574261 |
| 2.93597 | 0.394759 |
| 9.54734 | 0.959064 |
| 5.97708 | 0.543878 |
| 3.00956 | 0.412285 |
| 7.59119 | 0.914366 |
| 12.3221 | 0.976054 |
| 10.7388 | 0.931758 |
| 18.7786 | 0.942512 |
| 7.58839 | 0.890375 |
| 2.53858 | 0.417359 |
| 11.7775 | 0.962091 |
| 9.86204 | 0.925052 |
| 8.61377 | 0.894424 |
| 8.57032 | 0.841174 |
| 12.8429 | 0.942353 |
| 7.45663 | 0.90844 |
| 12.0221 | 0.921457 |
| 10.7767 | 0.90701 |
| 9.82209 | 0.943155 |
| 14.5979 | 0.989193 |
| 10.4613 | 0.922676 |
| 4.16601 | 0.432545 |
| 13.5907 | 0.960963 |
| 8.97963 | 0.923728 |
| 1.74799 | 0.359399 |
| 10.237 | 0.975335 |
| 13.3538 | 0.973928 |
| 8.0762 | 0.936685 |
| 13.3202 | 0.971127 |
| 10.4382 | 0.848878 |
| 9.89568 | 0.950542 |
| 12.0908 | 0.972157 |
| 13.672 | 0.966276 |
| 16.2323 | 0.956687 |
| 9.01397 | 0.911861 |
| 4.46669 | 0.72805 |
| 1.85732 | 0.255858 |
| 3.63264 | 0.427996 |
| 13.6496 | 0.982132 |
| 1.79565 | 0.269074 |
| 1.71434 | 0.231055 |
| 11.111 | 0.939893 |
| 4.2347 | 0.798951 |
| 8.93407 | 0.984258 |
| 2.7201 | 0.433258 |
| 15.148 | 0.95531 |
| 13.0223 | 0.936261 |
| 2.14889 | 0.501097 |
| 20.5756 | 0.989329 |
| 12.0859 | 0.953312 |
| 11.8602 | 0.949028 |
| 8.94809 | 0.931841 |
| 4.6398 | 0.775293 |
| 4.88791 | 0.549494 |
| 7.4356 | 0.783315 |
| 13.2676 | 0.978363 |
| 1.3611 | 0.282191 |
| 12.8583 | 0.98005 |
| 3.41467 | 0.469653 |
| 6.09342 | 0.780977 |
| 6.93167 | 0.834408 |
| 9.9146 | 0.958746 |
| 8.14489 | 0.847183 |
| 11.91 | 0.984841 |
| 7.37532 | 0.811862 |
| 8.99365 | 0.963317 |
| 12.0936 | 0.957535 |
| 11.6346 | 0.984796 |
| 6.84476 | 0.839395 |
| 12.7188 | 0.94865 |
| 5.69392 | 0.748557 |
| 16.4061 | 0.958867 |
| 11.6549 | 0.954228 |
| 14.3533 | 0.910211 |
| 4.46248 | 0.556505 |
| 8.26824 | 0.887567 |
| 7.20641 | 0.875609 |
| 9.37352 | 0.93007 |
| 7.48326 | 0.938433 |
| 2.7944 | 0.482628 |
| 13.7281 | 0.942822 |
| 4.70779 | 0.655398 |
| 12.1273 | 0.986339 |
| 11.3871 | 0.965489 |
| 9.00907 | 0.899555 |
| 12.0936 | 0.984614 |
| 3.36491 | 0.604916 |
| 14.5032 | 0.957353 |
| 11.214 | 0.956438 |
| 11.7074 | 0.98413 |
| 13.8598 | 0.980459 |
| 12.3663 | 0.973398 |
| 8.65302 | 0.970514 |
| 9.98048 | 0.920693 |
| 3.87865 | 0.528027 |
| 5.36942 | 0.722557 |
| 11.083 | 0.949195 |
| 10.8776 | 0.916046 |
| 10.0541 | 0.933075 |
| 10.3043 | 0.954894 |
| 13.5788 | 0.966004 |
| 7.41457 | 0.76357 |
| 12.5268 | 0.945615 |
| 11.65 | 0.964187 |
| 10.5945 | 0.914199 |
| 10.5356 | 0.945486 |
| 6.77748 | 0.784609 |
| 12.6754 | 0.933332 |
| 2.01993 | 0.282974 |
| 10.3744 | 0.950489 |
| 9.78774 | 0.986476 |
| 3.0411 | 0.446122 |
| 2.29467 | 0.4247 |
| 9.94264 | 0.965913 |
| 4.69587 | 0.658265 |
| 9.87255 | 0.980141 |

| BB-RMSD (Å) | 1gb1-RDC |
| --- | --- |
| 8.464 | 0.893342 |
| 13.873 | 0.943615 |
| 16.062 | 0.895141 |
| 11.556 | 0.905084 |
| 10.973 | 0.955638 |
| 9.669 | 0.91326 |
| 1.324 | 0.15414 |
| 8.226 | 0.840099 |
| 5.247 | 0.549075 |
| 2.309 | 0.240548 |
| 9.362 | 0.952997 |
| 8.641 | 0.917399 |
| 1.495 | 0.171619 |
| 7.831 | 0.804989 |
| 6.943 | 0.694283 |
| 6.241 | 0.747089 |
| 6.654 | 0.712063 |
| 2.199 | 0.168097 |
| 8.353 | 0.938587 |
| 3.477 | 0.611444 |
| 5.106 | 0.729724 |
| 10.426 | 0.875512 |
| 3.169 | 0.492069 |
| 9.992 | 0.946643 |
| 11.332 | 0.989801 |
| 8.489 | 0.942335 |
| 1.572 | 0.213961 |
| 11.592 | 0.917403 |
| 4.585 | 0.521714 |
| 10.556 | 0.939229 |
| 1.69 | 0.239629 |
| 17.862 | 0.929671 |
| 10.433 | 0.959778 |
| 14.491 | 0.964972 |
| 12.397 | 0.956684 |
| 7.199 | 0.872586 |
| 11.968 | 0.900165 |
| 3.813 | 0.697178 |
| 1.75 | 0.303464 |
| 6.715 | 0.779556 |
| 11.725 | 0.892415 |
| 6.903 | 0.528833 |
| 6.02 | 0.631486 |
| 1.35 | 0.188218 |
| 15.122 | 0.886269 |
| 4.867 | 0.704524 |
| 21.159 | 0.925013 |
| 8.866 | 0.806271 |
| 7.834 | 0.873398 |
| 11.03 | 0.936214 |
| 3.53 | 0.368446 |
| 4.536 | 0.57118 |
| 6.144 | 0.926783 |
| 14.581 | 0.901356 |
| 14.703 | 0.941547 |
| 13.098 | 0.851223 |
| 16.326 | 0.900007 |
| 0.92 | 0.0899621 |
| 7.054 | 0.795688 |
| 11.223 | 0.808337 |
| 11.296 | 0.824834 |
| 3.672 | 0.572586 |
| 4.692 | 0.708955 |
| 7.871 | 0.85507 |
| 13.195 | 0.954515 |
| 13.779 | 0.889065 |
| 10.395 | 0.757814 |
| 10.535 | 0.873575 |
| 1.223 | 0.164361 |
| 15.406 | 0.948615 |
| 1.372 | 0.158931 |
| 12.184 | 0.850523 |
| 11.266 | 0.864942 |
| 8.295 | 0.906755 |
| 5.412 | 0.657043 |
| 9.275 | 0.809911 |
| 3.281 | 0.508206 |
| 1.136 | 0.157492 |
| 1.32 | 0.18269 |
| 9.893 | 0.837151 |
| 6.394 | 0.463993 |
| 12.21 | 0.895092 |
| 1.771 | 0.153846 |
| 14.878 | 0.891117 |
| 4.957 | 0.547763 |
| 5.946 | 0.835829 |
| 12.711 | 0.965503 |
| 20.878 | 0.902922 |
| 8.375 | 0.839851 |
| 4.372 | 0.407548 |
| 10.072 | 0.915684 |
| 3.201 | 0.406285 |
| 1.543 | 0.146656 |
| 6.125 | 0.530525 |
| 1.074 | 0.12923 |
| 11.338 | 0.861625 |
| 9.749 | 0.92702 |
| 13.006 | 0.975705 |
| 6.625 | 0.866293 |
| 6.488 | 0.834238 |
| 9.707 | 0.799153 |
| 9.845 | 0.959804 |
| 12.426 | 0.850205 |
| 8.635 | 0.937486 |
| 13.213 | 0.932038 |
| 5.585 | 0.582702 |
| 19.054 | 0.947856 |
| 10.005 | 0.810734 |
| 5.734 | 0.6568 |
| 12.755 | 0.860007 |
| 2.104 | 0.281852 |
| 10.96 | 0.959103 |
| 4.874 | 0.423962 |
| 9.233 | 0.931764 |
| 10.677 | 0.919299 |
| 2.589 | 0.364508 |
| 2.369 | 0.221362 |
| 15.519 | 0.945914 |
| 4.792 | 0.639064 |
| 21.031 | 0.995559 |
| 13.093 | 0.88602 |
| 0.991 | 0.108249 |
| 11.435 | 0.815079 |
| 5.436 | 0.86028 |
| 13.096 | 0.920395 |
| 4.166 | 0.620125 |
| 1.111 | 0.12178 |
| 13.659 | 0.963098 |
| 11.915 | 0.759388 |
| 10.528 | 0.936297 |
| 7.602 | 0.912184 |
| 7.718 | 0.861252 |
| 15.537 | 0.847951 |
| 8.906 | 0.864349 |
| 6.459 | 0.887803 |
| 4.971 | 0.746717 |
| 11.043 | 0.897002 |
| 9.177 | 0.75103 |
| 10.549 | 0.710632 |
| 8.202 | 0.910916 |
| 3.031 | 0.443781 |
| 7.024 | 0.605998 |
| 7.276 | 0.867282 |
| 1.057 | 0.129258 |
| 8.18 | 0.833907 |
| 1.735 | 0.18232 |
| 9.906 | 0.956756 |
| 6.578 | 0.645533 |
| 15.683 | 0.819277 |
| 2.356 | 0.228969 |
| 4.216 | 0.565738 |
| 10.587 | 0.945747 |
| 7.465 | 0.84881 |
| 9.388 | 0.899644 |
| 14.029 | 0.973283 |
| 1.551 | 0.125946 |
| 10.132 | 0.960028 |
| 9.392 | 0.952153 |
| 3.266 | 0.377406 |
| 11.805 | 0.956782 |
| 11.345 | 0.977321 |
| 10.307 | 0.917564 |
| 11.188 | 0.944502 |
| 2.077 | 0.460653 |
| 14.885 | 0.929037 |
| 6.697 | 0.844201 |
| 2.234 | 0.568883 |
| 1.559 | 0.187117 |
| 17.427 | 0.938895 |
| 9.46 | 0.88647 |
| 1.739 | 0.164756 |
| 14.362 | 0.918381 |
| 11.586 | 0.788373 |
| 8.142 | 0.841199 |
| 10.251 | 0.833989 |
| 4.947 | 0.872049 |
| 11.77 | 0.896601 |
| 19.548 | 0.942964 |
| 6.897 | 0.553768 |
| 1.377 | 0.147678 |
| 15.93 | 0.851151 |
| 2.036 | 0.28668 |
| 7.444 | 0.95288 |
| 13.441 | 0.899841 |
| 8.889 | 0.931441 |
| 12.21 | 0.94885 |
| 1.461 | 0.169154 |
| 12.501 | 0.891952 |
| 11.852 | 0.932728 |
| 7.391 | 0.770633 |
| 9.064 | 0.970925 |
| 5.294 | 0.746964 |
| 4.298 | 0.548231 |
| 4.066 | 0.621436 |
| 8.348 | 0.837298 |
| 10.993 | 0.837346 |
| 10.347 | 0.894532 |
| 11.446 | 0.86486 |
| 3.105 | 0.596196 |
| 2.874 | 0.500654 |

| BB-RMSD (Å) | 2kdi-NOE |
| --- | --- |
| 8.94669 | 0.104963 |
| 14.9441 | 0.248285 |
| 12.0621 | 0.128458 |
| 9.84241 | 0.0683861 |
| 1.57487 | 0.00438513 |
| 8.99155 | 0.0841135 |
| 7.9178 | 0.0344604 |
| 8.11825 | 0.085719 |
| 8.4736 | 0.0627966 |
| 4.67765 | 0.0379936 |
| 10.5503 | 0.100424 |
| 9.70294 | 0.0806697 |
| 1.60851 | 0.00400155 |
| 4.24451 | 0.0110824 |
| 2.67735 | 0.00900736 |
| 3.54644 | 0.013969 |
| 4.17162 | 0.0169197 |
| 13.1316 | 0.205098 |
| 11.7334 | 0.259516 |
| 18.1177 | 0.449349 |
| 14.928 | 0.246963 |
| 10.7402 | 0.0924791 |
| 10.7164 | 0.140313 |
| 5.91189 | 0.0592321 |
| 3.7581 | 0.00874085 |
| 14.1935 | 0.222843 |
| 10.0394 | 0.0519694 |
| 11.5217 | 0.134869 |
| 4.33492 | 0.014457 |
| 11.8385 | 0.197393 |
| 12.7209 | 0.165486 |
| 14.8691 | 0.295249 |
| 12.8443 | 0.190237 |
| 11.4313 | 0.118987 |
| 3.79385 | 0.0194574 |
| 3.02989 | 0.00987263 |
| 9.21583 | 0.0857655 |
| 19.9441 | 0.680017 |
| 4.82413 | 0.0334953 |
| 19.532 | 0.546966 |
| 4.20736 | 0.020537 |
| 5.16266 | 0.0252213 |
| 10.2384 | 0.189522 |
| 9.68051 | 0.0970389 |
| 4.9566 | 0.0222323 |
| 9.23195 | 0.0742787 |
| 9.84381 | 0.122238 |
| 7.66619 | 0.073001 |
| 8.97893 | 0.0862254 |
| 12.2702 | 0.265794 |
| 6.10884 | 0.0302696 |
| 11.247 | 0.0690988 |
| 5.16266 | 0.0169067 |
| 10.2006 | 0.0813295 |
| 1.70944 | 0.00364014 |
| 12.9739 | 0.131942 |
| 11.111 | 0.0982426 |
| 2.32831 | 0.00621761 |
| 1.77041 | 0.00420752 |
| 10.8208 | 0.101995 |
| 11.1495 | 0.088495 |
| 13.4968 | 0.276804 |
| 1.97998 | 0.00596052 |
| 9.53963 | 0.204266 |
| 9.20742 | 0.127667 |
| 13.0924 | 0.243242 |
| 10.1094 | 0.109492 |
| 2.45797 | 0.00446021 |
| 8.82053 | 0.0749928 |
| 13.5767 | 0.226936 |
| 3.9144 | 0.011392 |
| 6.24411 | 0.0396026 |
| 12.1182 | 0.150068 |
| 6.33943 | 0.0567341 |
| 9.99871 | 0.176345 |
| 12.0018 | 0.233585 |
| 8.99365 | 0.129206 |
| 13.0587 | 0.229115 |
| 2.32831 | 0.00475653 |
| 2.91144 | 0.00473476 |
| 5.518 | 0.0341429 |
| 12.3929 | 0.16036 |
| 8.06288 | 0.0474473 |
| 4.16461 | 0.0220398 |
| 2.93597 | 0.0057215 |
| 9.54734 | 0.151522 |
| 5.97708 | 0.0263983 |
| 3.00956 | 0.0151905 |
| 7.59119 | 0.0418945 |
| 12.3221 | 0.293083 |
| 10.7388 | 0.112713 |
| 18.7786 | 0.695737 |
| 7.58839 | 0.0440838 |
| 2.53858 | 0.00791682 |
| 11.7775 | 0.128585 |
| 9.86204 | 0.113942 |
| 8.61377 | 0.149991 |
| 8.57032 | 0.0662707 |
| 12.8429 | 0.221181 |
| 7.45663 | 0.023531 |
| 12.0221 | 0.204512 |
| 10.7767 | 0.133284 |
| 9.82209 | 0.0530463 |
| 14.5979 | 0.335605 |
| 10.4613 | 0.138694 |
| 4.16601 | 0.0161321 |
| 13.5907 | 0.273558 |
| 8.97963 | 0.06958 |
| 1.74799 | 0.00527369 |
| 10.237 | 0.0844007 |
| 13.3538 | 0.273618 |
| 8.0762 | 0.0889724 |
| 13.3202 | 0.17584 |
| 10.4382 | 0.0458781 |
| 9.89568 | 0.0660999 |
| 12.0908 | 0.215098 |
| 13.672 | 0.392952 |
| 16.2323 | 0.323447 |
| 9.01397 | 0.0855523 |
| 4.46669 | 0.0227483 |
| 1.85732 | 0.0045572 |
| 3.63264 | 0.0120704 |
| 13.6496 | 0.314421 |
| 1.79565 | 0.00568763 |
| 1.71434 | 0.00433922 |
| 11.111 | 0.129928 |
| 4.2347 | 0.0152892 |
| 8.93407 | 0.11659 |
| 2.7201 | 0.011532 |
| 15.148 | 0.367564 |
| 13.0223 | 0.209174 |
| 2.14889 | 0.00712255 |
| 20.5756 | 0.788881 |
| 12.0859 | 0.217612 |
| 11.8602 | 0.166496 |
| 8.94809 | 0.0824506 |
| 4.6398 | 0.02434 |
| 4.88791 | 0.0194264 |
| 7.4356 | 0.0720161 |
| 13.2676 | 0.304004 |
| 1.3611 | 0.00292675 |
| 12.8583 | 0.232912 |
| 3.41467 | 0.00630149 |
| 6.09342 | 0.0319849 |
| 6.93167 | 0.0421911 |
| 9.9146 | 0.1472 |
| 8.14489 | 0.0793667 |
| 11.91 | 0.0917473 |
| 7.37532 | 0.0890264 |
| 8.99365 | 0.0586679 |
| 12.0936 | 0.133522 |
| 11.6346 | 0.153755 |
| 6.84476 | 0.0698368 |
| 12.7188 | 0.254258 |
| 5.69392 | 0.0638665 |
| 16.4061 | 0.540889 |
| 11.6549 | 0.216575 |
| 14.3533 | 0.207504 |
| 4.46248 | 0.0248892 |
| 8.26824 | 0.0880835 |
| 7.20641 | 0.0381561 |
| 9.37352 | 0.0761988 |
| 7.48326 | 0.0311279 |
| 2.7944 | 0.00954993 |
| 13.7281 | 0.291807 |
| 4.70779 | 0.0214577 |
| 12.1273 | 0.178008 |
| 11.3871 | 0.244814 |
| 9.00907 | 0.109031 |
| 12.0936 | 0.149508 |
| 3.36491 | 0.00854707 |
| 14.5032 | 0.249508 |
| 11.214 | 0.254626 |
| 11.7074 | 0.198251 |
| 13.8598 | 0.121884 |
| 12.3663 | 0.177816 |
| 8.65302 | 0.145311 |
| 9.98048 | 0.0858207 |
| 3.87865 | 0.0184056 |
| 5.36942 | 0.0273791 |
| 11.083 | 0.0963963 |
| 10.8776 | 0.106561 |
| 10.0541 | 0.120679 |
| 10.3043 | 0.0928291 |
| 13.5788 | 0.273844 |
| 7.41457 | 0.0217402 |
| 12.5268 | 0.233602 |
| 11.65 | 0.222027 |
| 10.5945 | 0.192999 |
| 10.5356 | 0.0975665 |
| 6.77748 | 0.0239199 |
| 12.6754 | 0.138997 |
| 2.01993 | 0.004716 |
| 10.3744 | 0.130933 |
| 9.78774 | 0.0624652 |
| 3.0411 | 0.013038 |
| 2.29467 | 0.00625392 |
| 9.94264 | 0.11315 |
| 4.69587 | 0.014961 |
| 9.87255 | 0.0907589 |

| BB-RMSD (Å) | 1gb1-NOE |
| --- | --- |
| 8.464 | 0.0738209 |
| 13.873 | 0.15083 |
| 16.062 | 0.521217 |
| 11.556 | 0.261561 |
| 10.973 | 0.174366 |
| 9.669 | 0.0872082 |
| 1.324 | 0.00182558 |
| 8.226 | 0.0792641 |
| 5.247 | 0.0262969 |
| 2.309 | 0.00528628 |
| 9.362 | 0.156444 |
| 8.641 | 0.11827 |
| 1.495 | 0.00402449 |
| 7.831 | 0.0584909 |
| 6.943 | 0.0794607 |
| 6.241 | 0.0475137 |
| 6.654 | 0.0216236 |
| 2.199 | 0.00254984 |
| 8.353 | 0.065544 |
| 3.477 | 0.0173881 |
| 5.106 | 0.0287857 |
| 10.426 | 0.0551996 |
| 3.169 | 0.0122063 |
| 9.992 | 0.107015 |
| 11.332 | 0.153946 |
| 8.489 | 0.137029 |
| 1.572 | 0.0013296 |
| 11.592 | 0.196168 |
| 4.585 | 0.0336068 |
| 10.556 | 0.162892 |
| 1.69 | 0.000783783 |
| 17.862 | 0.374768 |
| 10.433 | 0.206171 |
| 14.491 | 0.402867 |
| 12.397 | 0.240914 |
| 7.199 | 0.0792876 |
| 11.968 | 0.21558 |
| 3.813 | 0.0134158 |
| 1.75 | 0.0041804 |
| 6.715 | 0.0432728 |
| 11.725 | 0.0991385 |
| 6.903 | 0.0362039 |
| 6.02 | 0.0416683 |
| 1.35 | 0.00167721 |
| 15.122 | 0.276914 |
| 4.867 | 0.0258193 |
| 21.159 | 0.518896 |
| 8.866 | 0.0534128 |
| 7.834 | 0.0353058 |
| 11.03 | 0.0695643 |
| 3.53 | 0.00542268 |
| 4.536 | 0.0189504 |
| 6.144 | 0.02221 |
| 14.581 | 0.309863 |
| 14.703 | 0.323726 |
| 13.098 | 0.232819 |
| 16.326 | 0.431045 |
| 0.92 | 0.00111653 |
| 7.054 | 0.0369718 |
| 11.223 | 0.162767 |
| 11.296 | 0.322313 |
| 3.672 | 0.00714037 |
| 4.692 | 0.031791 |
| 7.871 | 0.0813718 |
| 13.195 | 0.185459 |
| 13.779 | 0.137271 |
| 10.395 | 0.078563 |
| 10.535 | 0.124305 |
| 1.223 | 0.00096271 |
| 15.406 | 0.231005 |
| 1.372 | 0.00173319 |
| 12.184 | 0.254796 |
| 11.266 | 0.102889 |
| 8.295 | 0.0774286 |
| 5.412 | 0.021243 |
| 9.275 | 0.0661163 |
| 3.281 | 0.0080654 |
| 1.136 | 0.000813693 |
| 1.32 | 0.00132883 |
| 9.893 | 0.116079 |
| 6.394 | 0.0562241 |
| 12.21 | 0.146199 |
| 1.771 | 0.0018337 |
| 14.878 | 0.343602 |
| 4.957 | 0.0208134 |
| 5.946 | 0.0421518 |
| 12.711 | 0.105555 |
| 20.878 | 0.45188 |
| 8.375 | 0.0978173 |
| 4.372 | 0.0176765 |
| 10.072 | 0.148421 |
| 3.201 | 0.00427153 |
| 1.543 | 0.00154751 |
| 6.125 | 0.0397812 |
| 1.074 | 0.00123783 |
| 11.338 | 0.170489 |
| 9.749 | 0.180716 |
| 13.006 | 0.19748 |
| 6.625 | 0.0201749 |
| 6.488 | 0.0157727 |
| 9.707 | 0.15803 |
| 9.845 | 0.113785 |
| 12.426 | 0.144971 |
| 8.635 | 0.111668 |
| 13.213 | 0.232559 |
| 5.585 | 0.0356804 |
| 19.054 | 0.547324 |
| 10.005 | 0.0886536 |
| 5.734 | 0.0412232 |
| 12.755 | 0.114133 |
| 2.104 | 0.00328402 |
| 10.96 | 0.143494 |
| 4.874 | 0.010596 |
| 9.233 | 0.095377 |
| 10.677 | 0.0936066 |
| 2.589 | 0.00763828 |
| 2.369 | 0.0122043 |
| 15.519 | 0.315772 |
| 4.792 | 0.0284724 |
| 21.031 | 0.585458 |
| 13.093 | 0.242235 |
| 0.991 | 0.000807624 |
| 11.435 | 0.155338 |
| 5.436 | 0.0364144 |
| 13.096 | 0.196477 |
| 4.166 | 0.0161241 |
| 1.111 | 0.00140357 |
| 13.659 | 0.23217 |
| 11.915 | 0.194653 |
| 10.528 | 0.0864486 |
| 7.602 | 0.05556 |
| 7.718 | 0.0959129 |
| 15.537 | 0.369574 |
| 8.906 | 0.0866981 |
| 6.459 | 0.0268725 |
| 4.971 | 0.00780042 |
| 11.043 | 0.156966 |
| 9.177 | 0.138418 |
| 10.549 | 0.166705 |
| 8.202 | 0.0647505 |
| 3.031 | 0.0143244 |
| 7.024 | 0.040348 |
| 7.276 | 0.035342 |
| 1.057 | 0.00121416 |
| 8.18 | 0.0585015 |
| 1.735 | 0.00255666 |
| 9.906 | 0.0852477 |
| 6.578 | 0.0615711 |
| 15.683 | 0.15237 |
| 2.356 | 0.00674368 |
| 4.216 | 0.023549 |
| 10.587 | 0.212351 |
| 7.465 | 0.038134 |
| 9.388 | 0.118753 |
| 14.029 | 0.319208 |
| 1.551 | 0.00260092 |
| 10.132 | 0.0543817 |
| 9.392 | 0.0643974 |
| 3.266 | 0.00943447 |
| 11.805 | 0.223433 |
| 11.345 | 0.234229 |
| 10.307 | 0.113261 |
| 11.188 | 0.209528 |
| 2.077 | 0.00486926 |
| 14.885 | 0.31654 |
| 6.697 | 0.0403387 |
| 2.234 | 0.00823716 |
| 1.559 | 0.00189436 |
| 17.427 | 0.487295 |
| 9.46 | 0.0752481 |
| 1.739 | 0.00154391 |
| 14.362 | 0.276862 |
| 11.586 | 0.0981883 |
| 8.142 | 0.053601 |
| 10.251 | 0.092912 |
| 4.947 | 0.0364021 |
| 11.77 | 0.196868 |
| 19.548 | 0.631766 |
| 6.897 | 0.0425056 |
| 1.377 | 0.000881627 |
| 15.93 | 0.197882 |
| 2.036 | 0.00155302 |
| 7.444 | 0.0524824 |
| 13.441 | 0.332453 |
| 8.889 | 0.080852 |
| 12.21 | 0.355023 |
| 1.461 | 0.00366182 |
| 12.501 | 0.243181 |
| 11.852 | 0.162346 |
| 7.391 | 0.0434324 |
| 9.064 | 0.195363 |
| 5.294 | 0.0321061 |
| 4.298 | 0.0115269 |
| 4.066 | 0.0196663 |
| 8.348 | 0.056372 |
| 10.993 | 0.109905 |
| 10.347 | 0.0829908 |
| 11.446 | 0.166774 |
| 3.105 | 0.0111073 |
| 2.874 | 0.00369806 |

Comment 2.10: The description of the REDCRAFT does not explain very clearly if there are any tunable parameters that need to be adjusted prior to REDCRAFT calculations (although it is mentioned that some modules have been disabled). If success of structure optimization depends on certain program settings, this is very important to mention. If not, this needs to be mentioned too.

Response 2.10: Our previous publications on REDCRAFT do describe some of the search parameters. In this report, we have fixed nearly all of those parameters, which are now included in the manuscript in the “Structure Calculation with REDCRAFT” section. Example shown below:

REDCRAFT^12,30,35,43,48,76,80,90,99^ was used to calculate the structure of proteins from RDC data with a standard depth search of 1000. Additional features such as Adaptive Decimation, minimization^35^ (used parameters: 3, 1, 1-end, 5000), and 4-bond LJ^35^ (threshold of 50) terms were included in all calculations.

Comment 2.11: In Tab. 1 and the surrounding discussion, the authors use values (like 1.189), ranges of values (1.9 - 2.5), lists of values (0.098, 0.12) and lists of ranges (0.01 - 0.046, 0.01 - 0.038, 0.01 - 0.026). They never explain the meaning of this notational diversity. I suppose it has to do with multiple conformers in the NMR structures and multiple alignment media.

Response 2.11: Thanks for this observation. We have added the text below to the results (and other section) section to better describe these number. In short, the range of numbers (reported as x-y) is to reflect the results obtained from different fragments of the protein because of the gap in the RDC data. The collection of numbers (reported as x,y) is to indicate the results in different alignment media (e.g. Q-Factors in medium 1, 2, or 3).

As explained above, in some cases backbone RDC data are missing for a portion of a protein. In such instance, REDCRAFT accommodates fragmented structure determination and therefore, the structural comparison to the target structure is reported as a range representing the combined BB-RMSD’s calculated for each fragment separately (columns 3 and 4). The fifth and six columns of Table 1 provide a quality of structural fitness to the RDC data as a Q-factor^32^ reported for each of the alignment media separately. When structure calculation is conducted in fragments (due to gaps in RDCs), the Q-factors reported for REDCRAFT structures (column 5) will consist of a list of ranges. Each item of the list (separated by a comma) reports the Q-factors for each alignment medium, while each range reports the minimum and maximum of Q-factors across all the fragments in a particular alignment medium.

Comment 2.12: Tab. 1 and Fig. 1 do not quite agree. According to Tab. 1, it is x-ray structure which is not always available (panel D in the plot), not an NMR structure as implied in the figure caption. It is unclear why panel E in the plot misses the NMR (green) structure.

Response 2.12: Thank you. We have corrected this error.

Comment 2.13: “By starting the calculation for the entire protein, the algorithm must contend with the maximum level of complexity from the start, which transforms the problem into a global optimization”. This may be a problem for RDCs, but it is not a problem for NOEs, which handle such global optimization task just fine.

Response 2.13: Agreed. Our interpretation of the figure (Figure 4 now) lead to the clear complementarity between the two sources of data. As noted in Comment 2.7, a more informed approach would utilize NOEs to navigate the complexities of structure determination while away from the actual structure. The reliance can be shifted more to RDCs as the search approaches the native structure since they exhibit more sensitivity to structural variation.

Comment 2.14: “Sequence specific backbone and side-chain NMR resonance assignments were determined using standard triple-resonance NMR experiments (Table S2)”. Table S2 does not say anything about the assignment experiments. Nowhere in the text have the authors mentioned the experiments used to measure RDCs in PF2048.1 (although protein expression and purification are described in some detail, as well as the preparation of aligned samples).

Response 2.14. Thank you for bringing this to our attention. We have added the following segments of text to address this point:

Sequence specific backbone and side-chain NMR resonance assignments were determined using standard double- and triple-resonance NMR experiments, including 2D [^1^H- ^15^N]-HSQC, 2D [^1^H-^13^C]-HSQC (aromatic region), 2D [^1^H-^13^C]-HSQC (aliphatic region), 3D HNCACB, 3D CBCAcoNH, 3D HNcoCA, 3D HNCA, 3D HNcaCO, 3D HNCO, 3D HBHAcoNH, 3D CCH-TOCSY, 3D ^15^N-edited TOCSY, and 3D simultaneous ^13^C-aromatic,^13^C-aliphatic,^15^N edited NOESY (t_m_ = 80 ms). Raw fid data for all of these spectra and peak lists for the NOESY spectrum are deposited in the BioMagResDatabase (BMRB ID 30494).

Data were acquired for the isotropic and the two aligned samples to provide a complete set of ^15^N-^1^HN, residual dipolar couplings. Data collection for the ^15^N IPAP-HSQC included 256 t1 points, and 2048 t2 points collected over 12 h. Residual dipolar couplings were calculated as the difference of the coupling measured in the aligned and isotropic conditions.

Comment 2.15: Wording:

“due to their planarity with one another” – due to their co-planarity

“both structures … exhibited high quality structures”

“large proteins (as large as 573)” – 573 residues

“RECRAFT’s approach”

“aqueous proteins”

“an excessive number of RDCs than necessary” – excessive number or more than necessary

“computational models … concluded the helical nature … and resulted in an ensemble of

structures”

One and the same program is referred as RPF and RFP

Response: We have made the suggested changes. Thank you!
