# Supplementary material for: REDCRAFT: A computational platform using residual dipolar coupling NMR data for determining structures of perdeuterated proteins in solution
Source: PLoS Comput Biol. 2021 Feb 1;17(2):e1008060. doi: 10.1371/journal.pcbi.1008060 (PMC7877757; doi:10.1371/journal.pcbi.1008060)
Supplement: Attachment — Submitted filename: ReviewCommentsRound2.v2.docx [file pcbi.1008060.s009.docx]

General Response: We very much appreciate the careful reading and extensive constructive comments made by both Reviewer #1 and Reviewer #2. Both appear to be experts in modeling of protein structures with NMR data, and the revisions made in response to these comments have greatly improved our manuscript. Thank you very much.

Reviewer #1:

I would like to start by thanking the authors for their extensive and careful responses to my many previous comments. This revised version of the manuscript is substantially better and clearer for the reader. The methods are also more clearly presented and the results much better organized. I would only subdivide the REDCRAFT methods section to highlight the new algorithms. Nevertheless, I am happy to recommend this work for publication.

Response 1: We again thank Reviewer #1 for constructive criticisms and suggestions. In Revision2, we have subdivided the REDCRAFT methods section to highlight the new algorithms, as suggested.

Reviewer #2:

The manuscript by Cole, Daigham, Liu, Montelione and Valafar talks about solving protein structures based on residual dipolar couplings (RDCs). The text of the manuscript has been thoroughly revised. Following the revisions, I have no problem with the structure of the paper and its main message. I would like to thank the authors for their thoughtful and constructive approach to my previous critique. Still, there are a couple of fairly significant issues that I would like to raise. In addition, there are quite a few minor glitches in the text. It appears that no one actually proof-read the text before resubmitting it to the journal, which is somewhat disappointing. In any event, the paper can now be published after some minor revisions. I trust that the authors can take care of the remaining issues (there is no need for me to re-review this manuscript).

Comment 1. Response 2.5. "In cases where significant differences are observed between NOE-based models and the corresponding X-ray structures, the X-ray structure coordinates are often found to be inconsistent with the NOE data. Hence, the X-ray crystal structure may be very accurate relative to what is present in the crystal, but not accurately reflect the dominant structure in solution."

I believe this is a misconception. X-ray coordinates provide a far more accurate representation of protein structure in solution than NMR coordinates. To make sure that this is so, let us take a random globular protein, where both X-ray structure (with resolution 1.8 A or better) and NOE-based structure are available. Let us consider proteins other than ubiquitin or GB1 (for which there are some extrardinarily accurate NOE structures), but a run-of-the-mill protein. Then let us fit a set of experimental solution-state RDCs to the X-ray structure and, separately, to the NOEbased structure. There is little doubt that X-ray structure should produce a much better Q-factor than NOE-based structure – thus signifying that X-ray structure is a much better model for protein in solution. One can also repeat the same exercise with J-couplings, chemical shifts and other solution-state observables.

Response 2.1: We again thank Reviewer #2 for productive and insightful suggestions, and for taking the time to carefully read and consider our manuscript.

We agree with the comment that, in the best circumstances, X-ray crystal structures are more accurate than NMR structures, and the hypothetical comparison for a 1.8 Å crystal structure of a well-ordered soluble protein is an excellent illustration of this point. However, it is well documented there are some proteins for which crystallization selects one of several energetically-accessible conformations present in solution; the conformation providing the best crystals need not be the dominant conformation in solution. There are numerous examples in the literature (a few cited below) in which both NMR and X-ray crystal structures are available for the exact same protein, where differences in conformation are observed, and where regions of the X-ray crystal structure do not fit the NMR data. In most cases this involves adjustments in domain orientations, or difference in conformations of surface loops involved in crystal packing. In other cases, segments which are largely disordered in solution are stabilized into unique conformations in the cryogenically-cooled crystal lattice, or crystallization conditions induce swapped dimers which are not observed for the corresponding dimer in solution.

Aside from these special cases in which crystallization may select less-populated conformers from the distribution present in solution, induce dimer “swapping”, or cause domain reorientations, the accuracy of any structure is also affected by data completeness and diffraction resolution. In our experience, comparing collections of solution NMR and crystal structure pairs (DOI: 10.1002/prot.21507; DOI: 10.1002/pro.2774), including using NMR structures for phase estimates by molecular replacement (DOI 10.1016/j.str.2011.04.005), we observe that good-quality NMR structures have rmsd of < 1 Å to the corresponding crystal structure (DOI: 10.1002/prot.21507; DOI: 10.1002/pro.2774), and accuracy comparable to 2.5 to 3.0 Å X-ray crystal structures; i.e. more accurate than a crystal structure modeled using for example 3.5 Å diffraction data.

These discussions are in fact outside the scope of this study, and we agree with Reviewer 2 that we can minimize discussion of this point without impacting the publishably of the current study. Accordingly, in principle we agree to the following revision of our previous response:

In cases where significant differences are observed between NOE-based models and the corresponding X-ray structures, the X-ray structure coordinates are sometimes found to be inconsistent with the NOE data (for example PDBID 1HNG-B and 1A64-A, which is dimeric in solution by not in the crystal structure). In these cases, the X-ray crystal structure may not accurately reflect the dominant structure in solution (DOI: 10.1002/prot.21507; DOI: 10.1002/pro.2774).

Comment 2. Let me use X-ray crystallography as an example to make another point. Conventional protocol to solve a crystal structure ends at the refinement step, where one obtains, say, crystallographic R of 0.21 and Rfree of 0.24. In principle, one could drive the refinement further and achieve, say, R=0.08. But there is a price to pay, since Rfree shoots to something like 0.36 (one can find some examples of this behavior in the early literature). This type of a situation is not good – in the case of (R=0.08, Rfree=0.36) the coordinates are actually overtightened and have lower quality than in the case of (R=0.21, Rfree=0.24). I imagine that something like this also happens with the RDCbased refinement. When the authors obtain Q factors of 0.06 to 0.09 – these are likely overtightened structures of somewhat inferior quality. It would be good to investigate this aspect using a system with some redundancy in the measured RDC dataset, so that Qfree can be formed and inspected.

Response 2.2: We fully agree with the potential for overfitting and agree that cross validation of RDC Q scores would be a valuable approach for further testing and development. In short, the issue of overfitting is mitigated with the use of conventional refinement steps using programs such as Xplor-NIH or CNS, especially in the presence of additional data. This subtle point has been clarified in the Discussion section. In fact, careful examination of our results (especially Table 2) will reveal a slight degradation in Q-factors following the refinement steps in Xplor-NIH. This is precisely the case of balancing two competing reporters of structural fitness.

Comment 3. Abstract. "Here, we describe the new features of the protein structure modeling program REDCRAFT and focus on the new Adaptive Decimation (AD) feature. The AD plays a critical role in improving the robustness of REDCRAFT to missing or noisy data, while allowing structure determination of larger proteins from less data". This leads one to think that AD is a centerpiece of the paper. After that it is barely mentioned at all throughout the rest of the paper (Introduction, Results, Discussion) – all the way to the Algorithms and Methods section, where it is actually described quite nicely. This produces a bit of a strange impression. The authors may want to somehow correct this – either briefly describe the AD method in the Introduction or, alternatively, tone down the abstract.

Response: We have added description of AD in the Introduction section.

Comment 4. Line 67. "Despite the changes that NMR spectroscopy has overcome over the years". Changes that NMR spectroscopy underwent (or challenges that NMR spectroscopy has overcome).

Response: Thank you, this has been corrected to read: “Despite the changes that NMR spectroscopy underwent…”

Comment 5. Lines 92 and 98. "continued use of the conventional optimization techniques", "continue to rely on the traditional optimization techniques" – repetitive.

Response: Thank you, these have been combined into the following on line 91-93: “While these programs address some of the shortcomings of the traditional approaches, their continued use of the conventional optimization techniques, such as Levenberg-Marquardt^29^ or gradient descent, prevent full utilization of the rich information content of the RDC data.”

Comment 6. The authors constantly use the term "legacy software" for NMR-structure-solving programs. The term "legacy" means "no longer under active development". Do the authors imply that none of NMR-structure-solving programs are under active development anymore…?

Response: Thank you for your observation. Clearly, many of the existing and mainstream NMR software are still under development. We have replaced the term “legacy” with “conventional” or “traditional” where appropriate.

Comment 7. Line 112 and throughout the text. "…can be obtained from perdeuterated proteins, namely backbone C’-N, N-HN, and C’-H RDCs". Does the latter mean C’-HN? This inconsistency persists throughout the text (and in Tab. 4 one can also find N-H notation alongside with N-HN, same in line 649; also one comes across H-Cα, which appears distinct from Hα-Cα).

Response: Thank you. This has been corrected throughout the text to be consistent nomenclature.

Comment 8. Line 163. "a Q-factors"

Response: Thank you. This has been corrected to “In summary, when using RDC data only, structures with Q-factors of over 0.5 are considered poorly fit structures,…” on Lines 160 -161.

Comment 9. Line 177. "An example of the convergence of the top 50 ensemble structures resulting from REDCRAFT calculation for GB1 is shown in Supplemental Fig S2. These represent the top 50 structures calculated for GB1 using this set of RDC data". The second sentence is redundant.

Response: Thank you. We have removed the second sentence and just kept the first with the reference to the supplemental figure.

Comment 10. Line 186. "these RDC data was reduced"

Response: Thank you. This has been corrected to: “For the purposes of this study, the set of RDCs was reduced to contain just N-C’ and N-H^N^ RDCs” on lines 183-184.

Comment 11. Table 1 and elsewhere. There are rmsd values reported with three digits, e.g. 1.121 Å. Should be rounded to 1.12.

Response: Thank you. We have corrected this to that it is consistent significant digits throughout.

Comment 12. Lines 247, 250. "Van der Waals" should be van der Waals.

Response: Thank you. This has been corrected in both instances.

Comment 13. Line 249. "The Q-factor RDCs" should be "The Q-factors for RDC"

Response: Thank you. This has been corrected in the text.

Comment 14. Line 256. "An example of the convergence of the top 50 ensemble structures resulting from REDCRAFT calculation for PF2048.1 is shown in Supplemental Fig S3. These represent the top 50 structures calculated for PF2048.1 using this set of RDC data". The second sentence seems redundant.

Response: Thank you. The second sentence has been removed to get rid of the duplication.

Comment 15. Line 305. "Structure calculation of large proteins by NMR spectroscopy is facilitated through perdeuteration of the sample protein suppress nuclear relaxation pathways…". Should be "perdeuteration of the sample protein, which suppresses"

Response: Thank you. This sentence has been corrected to: “Structure calculation of large proteins by NMR spectroscopy is facilitated through perdeuteration of the sample protein, which suppresses nuclear relaxation pathways and provides sharper linewidths.” On lines 299-301.

Comment 16. Line 322. "This is a significant achievement since in most cases, since such proteins must be perdeuterated …"

Response: Thank you. The second “since” has been deleted in the text.

Comment 17. Line 350. "Structural elucidation of proteins from RDCs using REDCRAFT has other pragmatic advantages. For instance, characterization of protein structure does not have to be restricted to the entire protein". You can solve protein fragments instead of an entire structure using any data (including NOEs) – this is not a "pragmatic advantage" of RDCs.

Response. We agree with this point of Reviewer 2. Although some NOE data is long range, involving interactions between segments, some of the NOE data is within segments of the protein structure, and under favorable cases can be used to determine the structure of individual segment. The text has been modified to call this a feature of REDCRAFT, rather than an advantage compared with NOE-based analysis.

Comment 18. Line 429. "Therefore, NOEs become relatively insensitive to structural variation when less than 5 Å from the native structure." The plot suggests more like 2 Å. Indeed, if it would have been 5 Å, then NOE-based structures would have an accuracy of about 5 Å. This is not the case – their true accuracy is more like 2 Å. For your information, that’s much worse than the accuracy of a reasonable X-ray structure with a resolution of, says, 1.8 Å – the accuracy in this case amounts to about 0.3 Å (please see my comment #1).

Response: We agree that the true accuracy of best-quality “NOE based structures” is typically about 1 to 2 Å; i.e. comparable to 2.5 to 3 Å crystal structures. However, such structures generally include more data than just NOEs. The backbone conformation is generally constrained by dihedral angle restraints based on chemical shift data, and in some cases hydrogen-bond restraints, which improve the structural accuracy.

Comment 19. Line 492. "Amide sides" should be "sites" 20. Section Evaluation, line 5

Response: Thank you, “sides” has been changed to “sites”.

Comment 20. "Our evaluation of REDCRAFT was conducted in three phases…". The order of the "phases" discussed in this section is not the same as in the actual text.

Response: Thank you, we have changed this text so that it mimics the order of the results reported in the Results section.

Comment 21. Both Tables 4 and 5 can be easily relegated to the SI.

Response: We thank this reviewer for the suggestion. In our opinion the presence of these tables in the body of the manuscript is critical since a reader will have to frequently reference these tables. Therefore, we respectfully pass this decision to the editor and the publisher.

Comment 22. Line 610. "The NxSC1 was pooled…". Why it is called NxSC1 here instead of PF2048.1?

Response: Thank you. This has been correct to PF2048.1.

Comment 23. Line 629. "RDC data suing the software package REDCAT". Those litigious RDC data…

Response: Indeed! Thank you. We have corrected this to “using”.
